# On the Role of Store-Operated Calcium Entry in Acute and Chronic Neurodegenerative Diseases

**DOI:** 10.3389/fnmol.2018.00087

**Published:** 2018-03-22

**Authors:** Agnese Secondo, Giacinto Bagetta, Diana Amantea

**Affiliations:** ^1^Division of Pharmacology, Department of Neuroscience, Reproductive and Odontostomatological Sciences, University of Naples Federico II, Napoli, Italy; ^2^Department of Pharmacy, Health and Nutritional Sciences, Section of Preclinical and Translational Pharmacology, University of Calabria, Cosenza, Italy

**Keywords:** Alzheimer’s disease, endoplasmic reticulum, Orai, Parkinson’s disease, SOCE, STIM, stroke, TRPC

## Abstract

In both excitable and non-excitable cells, calcium (Ca^2+^) signals are maintained by a highly integrated process involving store-operated Ca^2+^ entry (SOCE), namely the opening of plasma membrane (PM) Ca^2+^ channels following the release of Ca^2+^ from intracellular stores. Upon depletion of Ca^2+^ store, the stromal interaction molecule (STIM) senses Ca^2+^ level reduction and migrates from endoplasmic reticulum (ER)-like sites to the PM where it activates the channel proteins Orai and/or the transient receptor potential channels (TRPC) prompting Ca^2+^ refilling. Accumulating evidence suggests that SOCE dysregulation may trigger perturbation of intracellular Ca^2+^ signaling in neurons, glia or hematopoietic cells, thus participating to the pathogenesis of diverse neurodegenerative diseases. Under acute conditions, such as ischemic stroke, neuronal SOCE can either re-establish Ca^2+^ homeostasis or mediate Ca^2+^ overload, thus providing a non-excitotoxic mechanism of ischemic neuronal death. The dualistic role of SOCE in brain ischemia is further underscored by the evidence that it also participates to endothelial restoration and to the stabilization of intravascular thrombi. In Parkinson’s disease (PD) models, loss of SOCE triggers ER stress and dysfunction/degeneration of dopaminergic neurons. Disruption of neuronal SOCE also underlies Alzheimer’s disease (AD) pathogenesis, since both in genetic mouse models and in human sporadic AD brain samples, reduced SOCE contributes to synaptic loss and cognitive decline. Unlike the AD setting, in the striatum from Huntington’s disease (HD) transgenic mice, an increased STIM2 expression causes elevated synaptic SOCE that was suggested to underlie synaptic loss in medium spiny neurons. Thus, pharmacological inhibition of SOCE is beneficial to synapse maintenance in HD models, whereas the same approach may be anticipated to be detrimental to cortical and hippocampal pyramidal neurons. On the other hand, up-regulation of SOCE may be beneficial during AD. These intriguing findings highlight the importance of further mechanistic studies to dissect the molecular pathways, and their corresponding targets, involved in synaptic dysfunction and neuronal loss during aging and neurodegenerative diseases.

## Introduction

Calcium (Ca^2+^) is a ubiquitous second messenger involved in a number of cellular functions. Ca^2+^ ions are extruded from the cell or sequestered in intracellular compartments (stores) by energy-driven pumps that maintain cytosolic Ca^2+^ concentrations approximately 100,000-fold the extracellular space. As a consequence of this large concentration gradient, a rapid influx of Ca^2+^ occurs upon opening of ion channels located on the plasma membrane (PM) or on the membranes of intracellular stores, including the endoplasmic reticulum (ER) that is considered the major intracellular store. In both excitable and non-excitable cells, including those involved in the immune response, Ca^2+^ signals are generated by store-operated Ca^2+^entry (SOCE), namely the opening of PM Ca^2+^ channels following the release of Ca^2+^ from intracellular stores. Upon ER leak, SOCE determines ER Ca^2+^ refilling through the activation of two currents: the Ca^2+^ release-activated Ca^2+^ current (I_CRAC_), a non-voltage activated and inwardly rectifying current, highly selective for Ca^2+^, and the store-operated Ca^2+^ current (I_SOC_), a non-selective outward current with different biophysical features (Golovina et al., 2001; Trepakova et al., 2001; Strübing et al., 2003; Ma et al., 2015; Lopez et al., 2016). Although the electrophysiological properties of I_CRAC_ are known since decades (Hoth and Penner, 1993; Lewis and Cahalan, 1995), the molecular components underlying SOCE have been discovered more recently, with the identification of stromal interaction molecule (STIM) and Orai proteins (Liou et al., 2005; Roos et al., 2005; Zhang et al., 2005; Feske et al., 2006; Peinelt et al., 2006). In vertebrates, two different *STIM*-related genes have been described (Williams et al., 2001). STIM1 and STIM2 homologous are broadly and ubiquitously expressed in murine and human brain tissue, with a predominant expression of STIM2 (Kraft, 2015). More precisely, while STIM1 is most prominent in the cerebellum, STIM2 expression dominates in hippocampus and cortex (Lein et al., 2007; Uhlén et al., 2015). Upon depletion of Ca^2+^ store, STIM senses Ca^2+^ level reduction and migrates from ER-like sites to the PM where it activates Orai (Zhang et al., 2005), a tetraspanning protein forming a ion-conducting pore highly selective for Ca^2+^ (Feske et al., 2006; Peinelt et al., 2006; Prakriya et al., 2006). This PM channel exists in three different forms, Orai1, Orai2 and Orai3 exhibiting distinct inactivation and permeability properties (DeHaven et al., 2007; Lis et al., 2007). All three forms interact with STIM1 to produce SOCE, although Orai1 mediates a quantitatively much higher current than the other two forms (Putney, 2017). In particular, upon dissociation of Ca^2+^ from its EF-hand domains, STIM oligomerizes and translocates to ER-PM, where it forms large protein clusters referred to as *puncta* (Luik et al., 2006). Then, Orai1 is recruited to these *puncta* and promotes Ca^2+^ influx. Like its homolog, STIM2 mediates SOCE, although with slower activation kinetics and requiring a weaker depletion of ER Ca^2+^ stores to be activated (Brandman et al., 2007; Stathopulos et al., 2009). Despite the focus on STIM-regulated Orai channels for I_CRAC_-mediated SOCE, evidence is growing for STIM-operated transient receptor potential channel (TRPC)1 activity in mediating I_SOC_. Indeed, TRPC1 is recruited to the PM upon Orai/STIM complex formation from Rab4-vescicles (Liu et al., 2003; Ambudkar et al., 2007). Therefore, TRPC1-mediated outward current is subordinated to I_CRAC_. There are six TRPC proteins in humans (TRPC1, TRPC3–TRPC7), divided into two subfamilies, TRPC1/TRPC4/TRPC5 and TRPC3/TRPC6/TRPC7, based on biochemical and functional similarities. Search for SOCE partners led to the identification of several TRPCs, including TRPC1, TRPC3 and TRPC4 that are differently involved in this phenomenon. TRPC3 and TRPC4 can be activated by store depletion, while activation of TRPC5, TRPC6 and TRPC7 occurs via store-independent mechanisms (Ambudkar et al., 2007; Liu et al., 2007; Venkatachalam and Montell, 2007). Interestingly, TRPC form heteromeric structures involved in SOCE (Goel et al., 2002; Hofmann et al., 2002; Strübing et al., 2003), including endogenous TRPC1/TRPC3 in human parotid gland ductal cells (Liu et al., 2005) and rat hippocampal cell lines (Wu et al., 2004), TRPC1/TRPC5 in vascular smooth muscle (Wu et al., 2004), TRPC1/TRPC4 in endothelial cells (Sundivakkam et al., 2012) and TRPC1/TRPC3/TRPC7 in HEK293 cells (Zagranichnaya et al., 2005).

Both *in vitro* and *in vivo* studies, as well as evidence in human subjects with defects in SOCE have disclosed the pivotal involvement of Orai and STIM in several physiological and pathological conditions, including platelet function and hemeostasis (Varga-Szabo et al., 2008; Braun et al., 2009; Ahmad et al., 2011; Berna-Erro et al., 2016), neuronal excitability (Moccia et al., 2015), hypoxic/ischemic neuronal injury (Berna-Erro et al., 2009; Zhang et al., 2014), cardiac hypetrophy (Hulot et al., 2011; Saliba et al., 2015; Bénard et al., 2016), proliferation of vascular smooth muscle cells (Potier et al., 2009; Zhang et al., 2011; Jia et al., 2017), carcinogenesis (Yang et al., 2009; Chen et al., 2011; White, 2017). Of particular interest is the role of SOCE in the modulation of innate and adaptive immunity (Feske et al., 2006; Oh-Hora et al., 2008; Demaurex and Nunes, 2016; Vaeth et al., 2017). Indeed, store-operated Ca^2+^ current, I_CRAC_, was for the first time observed in mast cells and T lymphocytes (Parekh and Penner, 1997), whose activation is controlled by SOCE (Feske et al., 2001, 2005). In fact, recurrent infections take place in patients with mutations in *Orai1* and *Stim1* genes that abolish SOCE (Feske, 2011; Lacruz and Feske, 2015). Although immunodeficiency was mainly ascribed to T-cell dysregulation and the original initial work was focused on adaptive immunity, subsequent evidence demonstrated that SOCE also represents a major pathway for generating calcium signals in innate immune cells (Feske, 2011). Accordingly, calcium signaling has been implicated in numerous functions performed by phagocytes (e.g., macrophages, neutrophils and dendritic cells), including differentiation, maturation, migration, secretion and phagocytosis (Demaurex and Nunes, 2016). Although the present review mainly focusses on experimental data documenting the role of neuronal SOCE in neurodegenerative diseases, given the close interaction between the immune system and the brain during diverse pathological conditions (Amantea, 2016), the contribution of SOCE in immune cells could be envisaged and should deserve further investigation.

## SOCE in Stroke

Stroke is a major cause of death and long-term disability worldwide. Occlusion of a cerebral artery is the most common cause of brain ischemia, characterized by reduced cerebral blood flow, oxygen deprivation and activation of a cascade of events that ultimately lead to tissue damage. Over the past three decades, crucial findings from animal models have improved our understanding of the pathobiological mechanisms contributing to ischemic brain damage (Dirnagl and Endres, 2014). The blockade of cerebral circulation leads within seconds to cessation of neuronal electrical activity and within few minutes to disruption of energy state and ion homeostasis. It is widely accepted that a critical factor in determining neuronal death in stroke is the progressive accumulation of intracellular Ca^2+^ that, together with Na^+^ ions, can precipitate necrosis and apoptosis of vulnerable neurons (Kumar et al., 2014).

**Figure 1:**
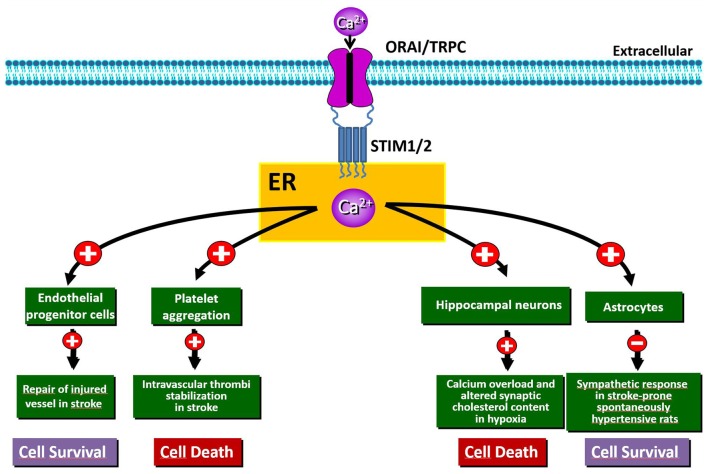
Schematic representation of the beneficial and/or detrimental effects of store-operated Ca^2+^ entry (SOCE) activation in different cell types involved in hypoxia/ischemia insult.

Despite this evidence, the mechanisms of Ca^2+^ cytotoxicity remain partially uncharacterized. In fact, Ca^2+^ seems to play a controversial role in this process since its increase or leak could assume different meanings. Accordingly, together with a detrimental role played by intracellular Ca^2+^ overload, it has been shown that Ca^2+^ leak from intracellular organelles can precipitate cell death (Pinton and Rizzuto, 2006). Thus, during brain ischemia, the complete depletion of Ca^2+^ from ER leads to neuronal death via ER stress (Ferri and Kroemer, 2001; Sirabella et al., 2009). Molecularly, the engagement of Gq-linked receptors results in a significant depletion of ER and in a robust influx of Ca^2+^ from the extracellular space that refills the organelle in order to reestablish Ca^2+^ homeostasis. This influx is specifically due to SOCE mediated by SOC channels and should protect neurons against ischemia-induced ER stress (Sirabella et al., 2009; Parekh, 2010). By contrast, in the hippocampus of rats exposed to global cerebral ischemia, the increased expression of STIM1 and Orai has been suggested to be the source of excessive calcium influx, whereby SOCE represents an important non-excitotoxic mechanism of ischemic neuronal death (Zhang et al., 2014). Thus, SOCE dysfunction is involved in stroke pathogenesis, but, to date, its exact role in brain ischemia is still controversial, since distinct mechanisms participating to either cell death or survival have been reported (Figure 1). Interestingly, SOCE has been widely investigated at the level of several components of the neurovascular unit, including astrocytes, endothelial cells and platelets, where it represents the major mechanism supporting agonist-induced Ca^2+^ signals (Rosado, 2016). By contrast, little is known about the functional importance of SOCE and its components at the neuronal level under both normoxic and hypoxic conditions.

### Role of STIM in Hypoxic/Ischemic Damage

Numerous *in vitro* studies have demonstrated that STIM2 is involved in ischemia-induced neuronal Ca^2+^ accumulation (Soboloff et al., 2006; Vig et al., 2006; Berna-Erro et al., 2009). In fact, hippocampal neurons both in culture and in acute hippocampal slices from *Stim2*^−/−^ mice show significantly reduced Ca^2+^ accumulation into ER during hypoxia and increased survival under hypoxic conditions compared to wild-type neurons (Berna-Erro et al., 2009). Accordingly, deficiency of STIM2 confers protection against stroke in mice (Berna-Erro et al., 2009). This is possibly due to the stabilization of cholesterol content at the level of neuronal PM (Sodero et al., 2012). In fact, the lack of STIM2 abolishes variations in synaptic cholesterol content in neurons in response to high excitatory neurotransmission (Sodero et al., 2012), a conditions occurring in stroke as well as in epilepsy and brain trauma (Müller and Connor, 1991).

Interestingly, wild-type mice reconstituted with *Stim2^−/−^* bone marrow developed infarcts like those of control wild-type mice, whereas infarcts were smaller in *Stim2*^−/−^ mice transplanted with wild-type bone marrow (Berna-Erro et al., 2009). This highlights that the neuroprotective effects exerted by STIM2 deficiency in mice are independent of functional alterations within the hematopoietic system. This is of interest since, in various immune cell types, such as T cells and phagocytes, SOCE controls a number of functions, including the release of cytokines, the production of reactive oxygen species, migration and polarization (Feske, 2007, 2011; Demaurex and Nunes, 2016). Regarding the immune cells of the brain, there is evidence that STIM1 and STIM2, modulating Orai1, regulate SOCE and purinergic activation of microglia (Ikeda et al., 2013; Michaelis et al., 2015). In fact, pharmacological inhibition of SOC channels reduces proinflammatory and neurotoxic responses of microglia (Lee et al., 2011). Given the crucial role of purinergic signaling in microglia activation following an ischemic insult (Pedata et al., 2016), it is intriguing to speculate that STIM and Orai may have a role in this process. Since activation of the innate and adaptive immune systems has been demonstrated to play a major role in the progression of cerebral damage (Amantea et al., 2014, 2015), investigating the relevance of SOCE in the modulation of immune cells following stroke is of pivotal importance.

Another component of the neurovascular unit that rapidly senses the reduction of blood flow is represented by astrocytes, whose function is critically affected by Ca^2+^ signaling. In these cells, hypoxia triggers Ca^2+^ release from ER stores, efficiently buffered by mitochondria (Smith et al., 2005). Such release of Ca^2+^ is sufficient to trigger capacitative Ca^2+^ entry. These findings indicate that the local O_2_ level is a key determinant of astrocyte Ca^2+^ signaling, likely modulating their Ca^2+^-dependent functions. Playing a pivotal role in neurovascular signaling in the brain (Haydon and Carmignoto, 2006; Viscomi et al., 2008; Mishra et al., 2016; Lu et al., 2017; Lind et al., 2018), astrocytes have also been implicated in the pathogenesis of hypertension in stroke-prone spontaneously hypertensive rat (SHRSP) via a sympatho-excitation mechanism (Allen et al., 2006; Isegawa et al., 2014). In particular, the reduction of glial *STIM1* in SHRSP has been identified as a candidate pathogenic mechanism responsible for this exaggerated sympathetic response leading to stroke. In fact, truncated STIM1 expressed in SHRSP astrocytes fails to interact with the PM Ca^2+^ channel TRPC1 and impairs SOCE function (Ohara and Nabika, 2016). Since the interaction with the C-terminus of STIM1 is required for TRPC1 activation (Huang et al., 2006), SOCE activity is decreased in astrocytes from SHRSP resulting in perturbation of downstream gene and neuronal network dysfunction. In the same direction are also the new strategies against the de-endothelialization of blood vessels, considered an early event of severe vascular disorders, including stroke. For instance, restoration of the endothelial lining may be accomplished by circulating endothelial progenitor cells (EPCs). In this context, SOCE activation favoring EPCs homing to the injured vessel is now considered the most appropriate route to therapeutically induce the regrowth of denuded vessels (Moccia et al., 2014).

### Role of Orai in Hypoxic/Ischemic Damage

It has been shown that the increased expression of ORAI1 and STIM1, occurring in the rat hippocampus after global ischemia, underlies detrimental intracellular Ca^2+^ accumulation, thus leading to neuronal death (Zhang et al., 2014). Moreover, deletion of *Orai1* in platelets results in resistance to ischemic brain infarction of mice subjected to transient middle cerebral artery occlusion (Braun et al., 2009). Analysis of global neurological function and of motor function and coordination revealed that *Orai1*^−/−^ mouse chimeras develop fewer neurological deficits compared with controls (Braun et al., 2009). This is consistent with the evidence that SOCE in platelets plays a crucial role in the stabilization of intravascular thrombi (Varga-Szabo et al., 2008). Besides, studies involving *Orai1* gain-of-function mutants in platelets indicate that SOCE might play a prominent role in thrombus formation, thus suggesting that the inhibition of platelet activation through pharmacological modulation of SOCE may represent an important strategy to prevent or treat acute ischemic events (Bhatt and Topol, 2003; van Kruchten et al., 2012). However, the role of Orai and its relationship with the other partners contributing to SOCE in stroke pathogenesis remains obscure; therefore, further work should be undertaken to clarify their specific function at the level of neurons, glia and hematopoietic cells.

## SOCE in Alzheimer’s Disease

Alzheimer’s disease (AD) is a neurodegenerative disorder that causes progressive memory loss, impairment of other cognitive functions and psychosis (Hort et al., 2010; McKhann et al., 2011). It represents the major cause of dementia worldwide and, in most cases, it appears sporadically after the age of 65 years (late-onset AD). The etiology of sporadic AD has not been clarified and its progression results from the combination of multiple factors (genetics, nutrition, lifestyle and chronic metabolic disorders), with aging being the major risk factor (Fjell et al., 2014; Scheltens et al., 2016). Less than 5% of cases correspond to early-onset AD, a familial form of the disease caused by genetic alterations, including mutations in genes encoding the amyloid-β precursor protein (APP), presenilin 1 (PSEN1) and presenilin 2 (PSEN2; Karch and Goate, 2015). PSEN1 and PSEN2 provide the catalytic subunit to the γ-secretase protease complex that, together with the β-secretase, catalyzes the sequential cleavage of APP to produce amyloid-β. The neuropathological features of both forms of AD comprise extraneuronal neuritic plaques mainly composed of insoluble amyloid-β_(40,42)_, intraneuronal neurofibrillary tangles consisting of hyperphosphorylated and misfolded tau, and synaptic/neuronal loss leading to brain atrophy (Serrano-Pozo et al., 2011). It was initially hypothesized that accumulation of insoluble amyloid-β in mature fibrils was an upstream contributor to the other pathological features; nevertheless, more recent findings have demonstrated that soluble amyloid-β_(1–42)_ oligomers play a more important role in neurotoxicity (Wang et al., 2017). Thus, inhibiting amyloid-β generation, facilitating its clearance and blocking oligomerization might represent promising strategies to block AD progression (Selkoe and Hardy, 2016). Nevertheless, the majority of these strategies failed in recent Phase III clinical trials (Godyn et al., 2016; Chen et al., 2017), supporting a poor association between brain amyloid-β levels and cognitive function (Doody et al., 2013; Briggs et al., 2017). Therefore, several key issues need to be reconsidered, most notably the pivotal role of amyloid-β in the upstream events that lead to neurodegeneration and the opportunity to target alternative pathways for clinical success.

Intriguingly, the earliest disclosed abnormality in AD pathogenesis, namely dysfunctional calcium signaling, is attracting renewed interest on the basis of its pivotal role in driving the synaptic defects occurring during memory loss (Khachaturian, 1989; Alzheimer’s Association Calcium Hypothesis Workgroup, 2017; Briggs et al., 2017). Accordingly, there is evidence that *PSEN* mutations, identified in familial AD, correlate with neuronal dysfunction and apoptosis through early dysregulation of Ca^2+^ homeostasis, that may occur before the appearance of amyloid-β or tau aggregation (Zeiger et al., 2013; Del Prete et al., 2014; Duggan and McCarthy, 2016). Therefore, the “calcium hypothesis of AD” strongly supports the concept that restoration of calcium homeostasis represents a valid alternative to targeting amyloid-β for the successful development of novel therapies.

Both in experimental models of AD and in patients, an excess release of Ca^2+^ from intracellular stores such as the ER was described (Pannaccione et al., 2012). In fact, in addition to γ-secretase function, PSENs also display a low conductance ER Ca^2+^ leak function through their hydrophilic catalytic cavity, and many familial AD *PSEN* mutations impair this function (Nelson et al., 2007, 2011). Regulation of Ca^2+^ levels by PSENs also involves their ability to interact with and activate Ca^2+^ channels: sarco/ER Ca^2+^-ATPase (SERCA) pump (Green et al., 2008), the inositol triphosphate receptor (IP3R; Cheung et al., 2008; Shilling et al., 2014) and the Ryanodine receptor (RyR; Stutzmann et al., 2007; Hayrapetyan et al., 2008). These effects of PSEN on Ca^2+^ may occur upstream of amyloid-β production and recent evidence also demonstrates that amyloid-β production is regulated by post-translational modifications of the RyR2 (Bussiere et al., 2017; Lacampagne et al., 2017). Accordingly, the RyR-mediated Ca^2+^ upregulation observed in synaptic compartments is associated with altered synaptic homeostasis and network depression at early, presymptomatic AD stages (Stutzmann et al., 2006; Chakroborty et al., 2009, 2012). Dysregulated RyR Ca^2+^ signaling disrupts synaptic function at both pre- and post-synaptic levels, thus affecting neuronal excitability and short and long term plasticity mechanisms implicated in learning and memory (Llano et al., 2000; Kuchibhotla et al., 2008; Chakroborty et al., 2012; Zhang et al., 2015a; Briggs et al., 2017).

The magnitude of Ca^2+^ signaling through IP3R and RyR channels is affected by the concentration of Ca^2+^ in the ER. Accordingly, it has been suggested that familial AD *PSEN* mutations may increase IP3R and RyR Ca^2+^ signaling through elevated ER Ca^2+^ stores. Although this hypothesis has been disputed (Bezprozvanny et al., 2012; Shilling et al., 2012), there is evidence that knockdown of *PSEN2* elevates ER Ca^2+^ stores corroborating the concept that unprocessed forms of PSEN function as ER Ca^2+^ leak channels (Nelson et al., 2007, 2011; Bandara et al., 2013). In addition, PSEN-1 M146V knockin (PSEN1KI) mutation disrupts ER Ca^2+^ leak functions of PSEN1, leading to ER Ca^2+^ elevation (Zhang et al., 2010; Sun et al., 2014), enhanced Ca^2+^-induced Ca^2+^-release from ER (Stutzmann et al., 2007; Zhang et al., 2010), increased activation of Ca^2+^-activated potassium channels and supranormal afterhyperpolarization (Stutzmann et al., 2006; Zhang et al., 2015a). These latter mechanisms lead to reduced excitability, synaptic depotentiation and impaired plasticity in KI neurons and may underlie memory defects (Zhang et al., 2015a).

**Figure 2:**
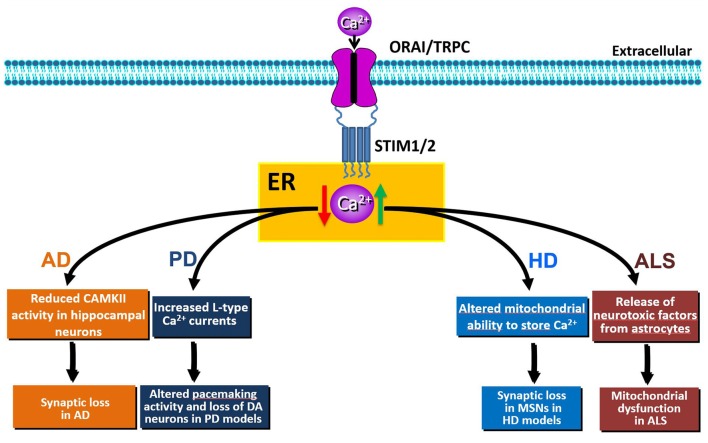
Schematic representation of the effects of SOCE dysfunction (elevation: green arrow or reduction: red arrow) in Alzheimer’s disease (AD), Huntington’s disease (HD), Parkinson’s disease (PD) or amyotrophic lateral sclerosis (ALS) pathobiology.

The movement of Ca^2+^ from the cytosol to the ER is controlled by SOCE and by SERCA pumps. In mice, both STIM1 and STIM2 are key regulators of synaptic plasticity in neural circuits encoding spatial learning and memory (Garcia-Alvarez et al., 2015). Moreover, a recent study has demonstrated that the maturation of dendritic spines and the formation of functional synapses in immature hippocampal neurons is facilitated by the influx of calcium through Orai1 (Korkotian et al., 2017). In hippocampal neurons from the PSEN1KI or the APP-knockin (APPKI) mouse models of AD and in human sporadic AD cortical samples, downregulation of STIM2 protein disrupts neuronal SOCE thus inhibiting the continuous Ca^2+^ influx necessary for sustained calmodulin kinase II (CaMKII) activation that is, in turn, essential for the stability of mushroom spines (Sun et al., 2014). In APPKI hippocampal neurons, extracellular amyloid-β42 over-activates mGluR5 receptor leading to elevated ER Ca^2+^ levels, compensatory downregulation of STIM2 expression, impairment of synaptic SOCE, and reduced CaMKII activity (Zhang et al., 2015b). Conversely, overexpression of STIM2 rescues neuronal SOCE and mushroom spine defects in hippocampal neurons from PS1KI and APPKI mouse models of familial AD (Sun et al., 2014; Zhang et al., 2015b) and protects mushroom spines from synaptotoxic effects of amyloid-β42 oligomers (Popugaeva et al., 2015). Thus, disturbance of STIM2-SOCE-CaMKII pathway contributes to synaptic loss and cognitive decline; conversely, upregulation of STIM2 expression levels or activation of synaptic neuronal SOCE pathway may yield therapeutic benefits for the treatment of AD and other age-related memory disorders (Figure 2).

Although in these latter studies the expression of STIM1 was not affected by aging or AD (Sun et al., 2014; Zhang et al., 2015b), recent studies identified STIM1 as a target of PSEN1-containing γ–secretase activity. In particular, PSEN1 mutations, associated with familial AD, enhanced γ–secretase cleavage of STIM1, reducing activation of Orai1 and attenuating SOCE (Tong et al., 2016). As a consequence of the inhibition of SOCE in hippocampal neurons, dendritic spine deformity was observed (Tong et al., 2016). Thus, PSEN-mediated cleavage of STIM1 may contribute to memory loss through SOCE dysregulation, and it has been hypothesized that STIM2 could also represent a target for γ-secretase, given the sequence similarities of the transmembrane domains of STIM1 and STIM2 (Tong et al., 2016). It is therefore intriguing to speculate that cleavage of STIM2 may account for the previously reported reduction of protein expression in AD (Sun et al., 2014; Zhang et al., 2015b).

The molecular identity of STIM-regulated neuronal SOCE in the spines is under investigation and recent findings have suggested that TRPCs may play a role. In fact, in addition to their classical individual effects, TRPC can complex with STIM or STIM/Orai to form SOCE (Ong et al., 2016). Of the seven members of the TRPC family, TRPC1 is the most extensively studied and has been involved in SOCE. However, in the settings where downregulation of STIM2 was detected, expression of TRPC1 was unaffected as no difference was found when comparing protein levels in brain samples from AD mice models or patients with controls (Sun et al., 2014). However, downregulation of TRPC1 and Orai1 proteins was suggested to underlie the reduction of SOCE in astrocytes from *APP* knockout mice (Linde et al., 2011; but also see, Ronco et al., 2014). Another member of the family that may also contribute to neuronal SOCE (Alkhani et al., 2014; Ong et al., 2016), TRPC3, was found to be negatively correlated with memory performance in mice (Neuner et al., 2015, 2017). By contrast, TRPC6 displays memory-preserving functions by promoting excitatory synapse formation through Ca^2+^-dependent pathways (Zhou et al., 2008) and/or by specifically interacting with APP thus leading to inhibition of its cleavage by γ-secretase and reduced amyloid-β production independently from its ion channel activity (Wang et al., 2015). Conversely, familial AD *PSEN2* mutations abolish agonist-induced TRPC6 activation and thus its Ca^2+^ entry function (Lessard et al., 2005). TRPC6 physically interacts with Orai (Liao et al., 2007) and TRPC6 and Orai2 channels form a STIM2-regulated neuronal SOCE channel complex in hippocampal mushroom spines (Zhang et al., 2016). Pharmacological activation of TRPC6 stimulates the activity of neuronal SOCE pathway in the spines and rescues mushroom spine loss and long-term potentiation impairment in the APP knockin mouse model of AD (Zhang et al., 2016). Thus, STIM2-regulated TRPC6/Orai2 SOCE channel complex in dendritic mushroom spines represents a promising therapeutic target for the treatment of memory loss in aging and AD.

An important point that needs to be addressed regards the evidence that downregulation of SOCE does not exclusively occur in neurons from different models of familial AD, but is also observed in astrocytes (Linde et al., 2011; Ronco et al., 2014), human microglia (Ronco et al., 2014) and in lymphoblasts obtained from familial AD patients (Bojarski et al., 2009). FAD mutations in endogenous PSEN1 in human B lymphocytes resulted in a decreased expression of STIM2 in parallel to an attenuation of capacitative Ca^2+^ entry (Bojarski et al., 2009). Thus, PSENs mutations may disrupt calcium homeostasis in various cell types and this may contribute to the multifactorial cascade that leads to neuronal dysfunction. Moreover, calcium signaling is also modified in peripheral cells obtained from sporadic AD patients (Ito et al., 1994; Gibson et al., 1996; Sulger et al., 1999). Lymphocytes from patients with mild cognitive impairment or sporadic AD exhibit an enhanced magnitude of calcium influx during SOCE, whereas, only the former were characterized by higher basal cellular calcium levels as compared to cells from non-demented subjects (Jaworska et al., 2013). Therefore, peripheral cells can be considered useful material for diagnosis and for drug screening.

The current treatment for AD relies on acetylcholinesterase inhibition (with donepezil, rivastigmine and galantamine) or blockade of NMDA receptor overstimulation (with memantine) that provide some symptomatic benefits for a limited period of time, followed by a steep cognitive decline due to the fact that these are not disease modifying drugs and do not prevent cell death. The current drug development pipeline is largely based on the amyloid hypothesis; however, despite efficacy at reducing amyloid levels in patients, all the clinical trials to date have generated disappointing results with respect to cognition function. Given the compelling evidence that aberrant calcium signaling, particularly in the ER, is associated with familial AD mutations and sporadic AD risk factors, modulation of calcium channels or calcium regulating proteins is in varying stages of therapeutic validation (Chakroborty and Stutzmann, 2014). In addition to targeting voltage-gated calcium channels, inhibition of RyR or restoration of SOCE have been suggested to represent promising approaches for drug development. Short-term treatment with the RyR inhibitor dantrolene stabilizes Ca^2+^ signals, ameliorates cognitive decline and reduces neuropathology, amyloid load and memory impairments in various AD mouse models (Chakroborty et al., 2012; Oulès et al., 2012; Peng et al., 2012). Regarding SOCE, its positive modulator NSN21778 rescues mushroom spine loss in both PSENKI and APPKI mouse models of AD (Zhang et al., 2016); while, its induction through overexpression of STIM2 or following pharmacological stimulation of TRPC6 is beneficial in AD animal models (Sun et al., 2014; Zhang et al., 2015b, 2016). Moreover, it is also noteworthy to highlight that, in addition to block NMDA receptor overstimulation, memantine increases SOCE in HEK cells (Blanchard et al., 2008). Thus, either through repositioned drugs or originally developed new molecular entities, therapeutic strategies aimed at promoting SOCE may represent promising ways for the development of effective AD therapies.

## Soce in Parkinson’s Disease

Parkinson’s disease (PD) is a neurodegenerative disorder characterized by the selective loss of dopaminergic (DA) neurons in the *substantia nigra pars compacta*. PD is mostly sporadic, but approximately 10–15% of cases are familial (Fleming, 2017). The major symptoms of PD involve motor dysfunctions, such as asymmetric bradykinesia, resting tremor, rigidity and postural instability (Ali and Morris, 2015). Pathogenic mechanisms of sporadic and familial PD are still elusive. However, the dysregulation of ER Ca^2+^ homeostasis is one of the mechanisms affecting the selective loss of DA neurons of the *substantia nigra pars compacta* (Stefani et al., 2012; Calì et al., 2014). Unlike other neurons, rhythmic activity of DA neurons depends on L-type Cav1.3 channels. Pharmacological inhibition of these currents by izradipine restores Ca^2+^−independent “juvenile” pacemaking activity and protects DA neurons in animal models of the disease (Chan et al., 2007). In normal conditions, the pacemaking activity of DA neurons is inhibited by the TRPC1-STIM1 complex. Accordingly, increased L-type Cav1.3 currents were observed upon *Stim1* or *TRPC1* silencing. Interestingly, the neurotoxin 1-methyl-4-phenylpyridinium ion (MPP+)—that mimics PD—decreases the level of TRPC1 and its interaction with STIM1, thus increasing neuronal death both *in vitro* and *in vivo* (Bollimuntha et al., 2005; Selvaraj et al., 2012). Molecularly, the decrease of TRPC1 expression leads to an abnormal increase in Cav1.3 activity, thereby causing degeneration of DA neurons (Sun et al., 2017; Figure 2). Despite the abnormal increase in L-type activity, downregulation of TRPC1 also leads to the loss of SOCE, thus triggering ER stress and initiation of the unfolded protein response (UPR) in DA neurons (Selvaraj et al., 2012). Conversely, in PC12 cell lines, *Stim1* knockdown significantly attenuated 6-hydroxydopamine (6-OHDA)- and MPP+-induced toxicity through inhibition of SOCE-mediated Ca^2+^-overload (Li et al., 2013, 2014); while, pharmacological inhibition of SOCE by SKF-96365 was protective against MPP+ cytotoxicity (Chen et al., 2013). The effect on SOCE was related to Orai1 and L-type Ca^2+^ channels, but not to TRPC1 (Li et al., 2014). Moreover, *Stim1* knockdown attenuated 6-OHDA- and MMP+-induced mitochondrial Ca^2+^ uptake and dysfunction in PC12 cells (Li et al., 2013, 2014). This further underscores that STIM1, through SOCE, may be responsible for neuronal oxidative stress induced by ER stress and mitochondrial dysfunction in PD.

In support of the important role of SOCE for DA neurons survival, the mutant dominant-negative form of Orai1 channel leads to tyrosine hydroxylase downregulation in *Drosophila* thus affecting dopamine synthesis and release (Pathak et al., 2015). Furthermore, skin fibroblasts from idiopathic PD patients and patients bearing familial R747W mutation in PLA2g6 gene, that encodes for a Ca^2+^- independent phospholipase A2, exhibit depleted stores and reduced SOCE (Zhou et al., 2016). Overall, these findings indicate that SOCE pathway in DA neurons represents an attractive target for PD drug discovery (Pchitskaya et al., 2018).

## Soce in Huntington’s Disease

HD is a fully penetrant neurodegenerative disorder, characterized by cognitive, motor and psychiatric disturbances, and caused by a dominantly inherited CAG trinucleotide repeat expansion in the huntingtin gene on chromosome 4 (McColgan and Tabrizi, 2018). Mutant huntingtin (mHtt) causes defective striatal neuron function, reduction of synaptic contacts, disruption of immune cell function and eventual neurodegeneration through a number of mechanisms (Kim and Fung, 2014; Andre et al., 2016).

Accumulating evidence indicates that dysregulation of intracellular neuronal Ca^2+^ signaling by mHTT plays a role in HD progression (Pchitskaya et al., 2018; Raymond, 2017). Unlike the AD setting, in the striatum from HD transgenic mice, an increased STIM2 expression causes elevated synaptic SOCE, that was suggested to underlie synaptic loss in medium spiny neurons (MSNs; Wu et al., 2011, 2016). After binding IP3R1, mHtt increases its sensitivity to activation by IP3, thus producing a reduction of ER Ca^2+^ levels that, in turn, promotes sustained and synaptotoxic SOCE in the spines of MSNs from YAC128 transgenic HD mice (Tang et al., 2005, 2009; Wu et al., 2016). In this context, STIM2 expression is elevated, as documented both in aged YAC128 striatal cultures and in YAC128 mouse striatum. In human neuroblastoma cells, the expression of the N-terminal fragment of mHtt (Htt138Q-1exon) is sufficient to cause a HD pathological phenotype, as it induces an increase of SOCE that requires STIM1 in this *in vitro* model (Vigont et al., 2014). Similar findings were obtained in mouse neuroblastoma cells and in primary culture of mouse MSNs, where lentiviral expression of Htt138Q-1exon results in enhanced SOCE through the involvement of the sensor STIM1 and of both TRPC1- and Orai1 subunits forming a heteromeric channel (Vigont et al., 2015). A key role of TRPC1 channels in supporting SOCE was also demonstrated in YAC128 MSNs primary cultures, where TRPC1 suppression by a short interfering RNA has a significant protective effect against glutamate-induced apoptosis (Wu et al., 2011).

Notably, pharmacological inhibition of neuronal SOCE by EVP4593 rescues striatal spine loss in both *in vitro* and *in vivo* transgenic HD models (Vigont et al., 2015; Wu et al., 2016); analogously, stabilization of SOCE by tetrahydrocarbazoles was associated with beneficial effects on the function of mitochondria from YAC128 MSNs (Czeredys et al., 2017).

Another mechanism that contributes to the dysregulation of SOCE during HD progression involves the sigma-1 receptor (S1R), an ER resident transmembrane protein that is regulated by ER Ca^2+^ homeostasis. Consistent with ER calcium dysregulation in HD, striatal upregulation of S1R occurs in aged YAC128 transgenic HD mice and in patients. Pharmacological activation of S1R with pridopidine prevents MSN spine loss in aging YAC128 co-cultures through suppression of supranormal ER Ca^2+^ release, restored ER calcium levels and reduced excessive SOCE in spines (Ryskamp et al., 2017).

Thus, pharmacological inhibition of SOCE is beneficial to synapse maintenance in HD models, whereas the same approach may be anticipated to be detrimental to cortical and hippocampal pyramidal neurons (Figure 2). Conversely, while up-regulation of SOCE may be beneficial for pyramidal neurons during AD, it may be detrimental for other CNS synapses. These intriguing findings highlight the importance of further mechanistic studies to dissect the molecular pathways, and their corresponding targets, involved in synaptic loss during aging and neurodegenerative diseases.

## Soce in Motor and Sensory Peripheral Nerve Degeneration

Amyotrophic lateral sclerosis (ALS) is a severe human progressive neurodegenerative disease affecting lower and upper motor neurons whose specific etiology is incompletely understood. Familial ALS (fALS) patients with known genetic mutations are relatively rare, and over 90% of cases occur sporadically. However, mutations in superoxide dismutase-1 (SOD1), TAR DNA-binding protein 43 (TARDBP/TDP-43) and C9orf72 have been identified in subsets of familial and sporadic patients (Rosen et al., 1993; DeJesus-Hernandez et al., 2011; Renton et al., 2011). In these ALS forms, dysregulation of calcium homeostasis, oxidative stress, ER stress and UPR have been identified as molecular and neuropathological features (Kaus and Sareen, 2015). Beside the evidence that perturbations in ER Ca^2+^ homeostasis are implicated in ALS pathology, data on the involvement of SOCE and its molecular components are currently limited. Some evidence on SOCE dysregulation in ALS was provided only in glial cells that actively participate to the neurodegenerative process of the disease. In particular, astrocytes obtained from animal models of ALS or from human familial or sporadic ALS patients are able to release toxic factors that induce cell death in primary motor neurons (Di Giorgio et al., 2007; Nagai et al., 2007; Haidet-Phillips et al., 2011). However, the mechanisms underlying this pathological release remain unclear. In this respect, it has been reported that mutant SOD1G93A astrocytes display aberrant ER calcium filling that involves enhanced S-glutathionylation of STIM1. This reversible posttranslational modification occurs in response to oxidative stress in a number of neurodegenerative diseases (Mieyal et al., 2008), including ALS (Poon et al., 2005; Cassina et al., 2008). STIM1 glutathionylation in the ER promotes its interaction with PM Orai1 in a calcium-independent manner thus inducing constitutive calcium entry (Hawkins et al., 2010). Increased STIM1 glutathionylation may, indeed, underlie continuous SOCE and ER calcium overload in SOD1G93A astrocytes (Kawamata et al., 2014).

SOCE dysfunction is also implicated in Charcot-Marie-Tooth (CMT) disease, one of the most frequent inherited neurological disorders characterized by either demyelinating or axonal neuropathy of motor and sensory peripheral nerves (Pareyson et al., 2013). Mutations in ganglioside-induced differentiation-associated protein-1 (GDAP1), which maps at human chromosome 8q21.1, are causative for autosomal recessive demyelinating CMT4A (Baxter et al., 2002). The severe recessive form of GDAP1-related CMT starts early in infancy or childhood with weakness and wasting of the feet followed by involvement of the hands leading to pronounced disability. In the animal models of CMT, mutations in the GDAP1 gene recapitulates pathological features of the disease causing a peripheral neuropathy with a loss of motor neurons and abnormal neuromuscular junctions. GDAP1 is located in the mitochondrial outer membrane where it participates in the mitochondrial network dynamics and in the mitochondria/ER coupling. Importantly, GDAP1 silencing significantly reduces Ca^2+^ inflow through SOCE upon mobilization of ER Ca^2+^. This causes a partial depletion of intracellular Ca^2+^ stores and/or release defects (Barneo-Muñoz et al., 2015). This suggests that GDAP1-related CMT neuropathy may be associated with a decrease in SOCE activity and impaired SOCE-driven Ca^2+^ uptake in intracellular storing organelles with a consequent abnormal distribution and movement of mitochondria throughout cytoskeleton towards the ER and subplasmalemmal microdomains (Pla-Martín et al., 2013).

## Conclusion

The experimental evidence reviewed in the present work clearly demonstrates that Ca^2+^ homeostasis and, most notably, SOCE components are dysregulated in both acute and chronic neurodegenerative diseases. Thus, SOCE is not only involved in Ca^2+^ refilling of ER, but also provides an influx of Ca^2+^ that performs signaling functions and may (positively or negatively) contribute to the progression of neurodegenerative diseases (Majewski and Kuznicki, 2015). In fact, SOCE and its components, namely STIM, Orai and TRPC, have a well-established significance in both excitable and non-excitable cells, including neurons, glial cells and leukocytes. Dysregulation of SOCE in these cells may play specific roles in neuronal demise or protection, depending on the nature of the insult (Figures 1, 2). SOCE seems to be neuroprotective in PD and AD, while in other situations, such as for instance HD, neuroprotection is achieved by blocking SOCE. The role of SOCE in stroke is instead still controversial. Dissection of the molecular components sustaining SOCE should be performed at the level of each specific cell type in order to identify the exact role played by SOCE in each of them. The dualistic role of SOCE in the different forms of neurodegeneration should be carefully considered when its elements are regarded as potential targets for the development of new drugs. Promising pharmacological targets for the modulation of SOCE are: the Ca^2+^ sensors (e.g., STIMs), the Ca^2+^ channels (e.g., Orais and TRPCs) or the proteins that sense Ca^2+^ entry through SOCE and then activate the cellular response. Although some compounds that modulate SOCE have been developed and tested in pre-clinical models of neurodegenerative diseases, the validation of new drugs that specifically target SOCE components would result in successful treatment strategies for currently incurable neurodegenerative diseases, including AD, PD, HD and stroke.

## Author Contributions

AS, GB and DA participated in the collection, review and analysis of the relevant literature, as well as in drafting and revising the manuscript.

## Conflict of Interest Statement

The authors declare that the research was conducted in the absence of any commercial or financial relationships that could be construed as a potential conflict of interest.

## References

[B1] Ahmad F. Boulaftali Y. Greene T. K. Ouellette T. D. Poncz M. Feske S.. (2011). Relative contributions of stromal interaction molecule 1 and CalDAG-GEFI to calcium-dependent platelet activation and thrombosis. J. Thromb. Haemost. 9, 2077–2086. 10.1111/j.1538-7836.2011.04474.x 21848641 PMC3184355

[B2] Ali K. Morris H. R. (2015). Parkinson’s disease: chameleons and mimics. Pract. Neurol. 15, 14–25. 10.1136/practneurol-2014-000849 25253895

[B3] Alkhani H. Ase A. R. Grant R. O’Donnell D. Groschner K. Séguéla P. (2014). Contribution of TRPC3 to store-operated calcium entry and inflammatory transductions in primary nociceptors. Mol. Pain 10, 1744–8069-10–43. 10.1186/1744-8069-10-43 24965271 PMC4118315

[B4] Allen A. M. Dosanjh J. K. Erac M. Dassanayake S. Hannan R. D. Thomas W. G. (2006). Expression of constitutively active angiotensin receptors in the rostral ventrolateral medulla increases blood pressure. Hypertension 47, 1054–1061. 10.1161/01.HYP.0000218576.36574.54 16618838

[B5] Alzheimer’s Association Calcium Hypothesis Workgroup. (2017). Calcium hypothesis of Alzheimer’s disease and brain aging: a framework for integrating new evidence into a comprehensive theory of pathogenesis. Alzheimers Dement. 13, 178.e17–182.e17. 10.1016/j.jalz.2016.12.006 28061328

[B6] Amantea D. (2016). Editorial overview: neurosciences: brain and immunity: new targets for neuroprotection. Curr. Opin. Pharmacol. 26, v–viii. 10.1016/j.coph.2015.12.001 26740370

[B7] Amantea D. Micieli G. Tassorelli C. Cuartero M. I. Ballesteros I. Certo M.. (2015). Rational modulation of the innate immune system for neuroprotection in ischemic stroke. Front. Neurosci. 9:147. 10.3389/fnins.2015.00147 25972779 PMC4413676

[B8] Amantea D. Tassorelli C. Petrelli F. Certo M. Bezzi P. Micieli G.. (2014). Understanding the multifaceted role of inflammatory mediators in ischemic stroke. Curr. Med. Chem. 21, 2098–2117. 10.2174/0929867321666131227162634 24372219

[B9] Ambudkar I. S. Ong H. L. Liu X. Bandyopadhyay B. Cheng K. T. Cheng K. T. (2007). TRPC1: the link between functionally distinct store-operated calcium channels. Cell Calcium 42, 213–223. 10.1016/j.ceca.2007.01.013 17350680

[B10] Andre R. Carty L. Tabrizi S. J. (2016). Disruption of immune cell function by mutant huntingtin in Huntington’s disease pathogenesis. Curr. Opin. Pharmacol. 26, 33–38. 10.1016/j.coph.2015.09.008 26461267

[B11] Bandara S. Malmersjö S. Meyer T. (2013). Regulators of calcium homeostasis identified by inference of kinetic model parameters from live single cells perturbed by siRNA. Sci. Signal. 6:ra56. 10.1126/scisignal.2003649 23838183 PMC3897207

[B12] Barneo-Muñoz M. Juárez P. Civera-Tregón A. Yndriago L. Pla-Martin D. Zenker J.. (2015). Lack of GDAP1 induces neuronal calcium and mitochondrial defects in a knockout mouse model of charcot-marie-tooth neuropathy. PLoS Genet. 11:e1005115. 10.1371/journal.pgen.1005115 25860513 PMC4393229

[B13] Baxter R. V. Ben Othmane K. Rochelle J. M. Stajich J. E. Hulette C. Dew-Knight S.. (2002). Ganglioside-induced differentiation-associated protein-1 is mutant in charcot-marie-tooth disease type 4A/8q21. Nat. Genet. 30, 21–22. 10.1038/ng796 11743579

[B14] Bénard L. Oh J. G. Cacheux M. Lee A. Nonnenmacher M. Matasic D. S.. (2016). Cardiac Stim1 silencing impairs adaptive hypertrophy and promotes heart failure through inactivation of mTORC2/Akt signaling. Circulation 133, 1458–1471; discussion 1471. 10.1161/CIRCULATIONAHA.115.020678 26936863 PMC4829441

[B15] Berna-Erro A. Braun A. Kraft R. Kleinschnitz C. Schuhmann M. K. Stegner D.. (2009). STIM2 regulates capacitive Ca^2+^ entry in neurons and plays a key role in hypoxic neuronal cell death. Sci. Signal. 2:ra67. 10.1126/scisignal.2000522 19843959

[B16] Berna-Erro A. Jardín I. Smani T. Rosado J. A. (2016). Regulation of platelet function by Orai, STIM and TRP. Adv. Exp. Med. Biol. 898, 157–181. 10.1007/978-3-319-26974-0_8 27161229

[B17] Bezprozvanny I. Supnet C. Sun S. Zhang H. De Strooper B. (2012). Response to Shilling et al. (10.1074/jbc.M111.300491). J. Biol. Chem. 287:20469, author reply 20470. 10.1074/jbc.L112.356790 22685228 PMC3370228

[B18] Bhatt D. L. Topol E. J. (2003). Scientific and therapeutic advances in antiplatelet therapy. Nat. Rev. Drug Discov. 2, 15–28. 10.1038/nrd985 12509756

[B19] Blanchard A. P. Guillemette G. Boulay G. (2008). Memantine potentiates agonist-induced Ca^2+^ responses in HEK 293 cells. Cell. Physiol. Biochem. 22, 205–214. 10.1159/000149798 18769047

[B20] Bojarski L. Pomorski P. Szybinska A. Drab M. Skibinska-Kijek A. Gruszczynska-Biegala J.. (2009). Presenilin-dependent expression of STIM proteins and dysregulation of capacitative Ca^2+^ entry in familial Alzheimer’s disease. Biochim. Biophys. Acta 1793, 1050–1057. 10.1016/j.bbamcr.2008.11.008 19111578

[B21] Bollimuntha S. Singh B. B. Shavali S. Sharma S. K. Ebadi M. (2005). TRPC1-mediated inhibition of 1-methyl-4-phenylpyridinium ion neurotoxicity in human SH-SY5Y neuroblastoma cells. J. Biol. Chem. 280, 2132–2140. 10.1074/jbc.M407384200 15542611 PMC3619406

[B22] Brandman O. Liou J. Park W. S. Meyer T. (2007). STIM2 is a feedback regulator that stabilizes basal cytosolic and endoplasmic reticulum Ca^2+^ levels. Cell 131, 1327–1339. 10.1016/j.cell.2007.11.039 18160041 PMC2680164

[B23] Braun A. Varga-Szabo D. Kleinschnitz C. Pleines I. Bender M. Austinat M.. (2009). Orai1 (CRACM1) is the platelet SOC channel and essential for pathological thrombus formation. Blood 113, 2056–2063. 10.1182/blood-2008-07-171611 18832659

[B24] Briggs C. A. Chakroborty S. Stutzmann G. E. (2017). Emerging pathways driving early synaptic pathology in Alzheimer’s disease. Biochem. Biophys. Res. Commun. 483, 988–997. 10.1016/j.bbrc.2016.09.088 27659710 PMC5303639

[B25] Bussiere R. Lacampagne A. Reiken S. Liu X. Scheuerman V. Zalk R.. (2017). Amyloid β production is regulated by β2-adrenergic signaling-mediated post-translational modifications of the ryanodine receptor. J. Biol. Chem. 292, 10153–10168. 10.1074/jbc.M116.743070 28476886 PMC5473221

[B26] Calì T. Ottolini D. Brini M. (2014). Calcium signaling in Parkinson’s disease. Cell Tissue Res. 357, 439–454. 10.1007/s00441-014-1866-0 24781149

[B27] Cassina P. Cassina A. Pehar M. Castellanos R. Gandelman M. de Leon A.. (2008). Mitochondrial dysfunction in SOD1G93A-bearing astrocytes promotes motor neuron degeneration: prevention by mitochondrial-targeted antioxidants. J. Neurosci. 28, 4115–4122. 10.1523/JNEUROSCI.5308-07.2008 18417691 PMC3844766

[B29] Chakroborty S. Briggs C. Miller M. B. Goussakov I. Schneider C. Kim J.. (2012). Stabilizing ER Ca^2+^ channel function as an early preventative strategy for Alzheimer’s disease. PLoS One 7:e52056. 10.1371/journal.pone.0052056 23284867 PMC3528716

[B30] Chakroborty S. Goussakov I. Miller M. B. Stutzmann G. E. (2009). Deviant ryanodine receptor-mediated calcium release resets synaptic homeostasis in presymptomatic 3xTg-AD mice. J. Neurosci. 29, 9458–9470. 10.1523/JNEUROSCI.2047-09.2009 19641109 PMC6666542

[B28] Chakroborty S. Stutzmann G. E. (2014). Calcium channelopathies and Alzheimer’s disease: insight into therapeutic success and failures. Eur. J. Pharmacol. 739, 83–95. 10.1016/j.ejphar.2013.11.012 24316360

[B31] Chan C. S. Guzman J. N. Ilijic E. Mercer J. N. Rick C. Tkatch T.. (2007). “Rejuvenation” protects neurons in mouse models of Parkinson’s disease. Nature 447, 1081–1086. 10.1038/nature05865 17558391

[B34] Chen Y.-F. Chiu W.-T. Chen Y.-T. Lin P.-Y. Huang H.-J. Chou C.-Y.. (2011). Calcium store sensor stromal-interaction molecule 1-dependent signaling plays an important role in cervical cancer growth, migration and angiogenesis. Proc. Natl. Acad. Sci. U S A 108, 15225–15230. 10.1073/pnas.1103315108 21876174 PMC3174613

[B32] Chen G. Xu T. Yan Y. Zhou Y. Jiang Y. Melcher K.. (2017). Amyloid β: structure, biology and structure-based therapeutic development. Acta Pharmacol. Sin. 38, 1205–1235. 10.1038/aps.2017.28 28713158 PMC5589967

[B33] Chen T. Zhu J. Zhang C. Huo K. Fei Z. Jiang X. (2013). Protective effects of SKF-96365, a non-specific inhibitor of SOCE, against MPP+-induced cytotoxicity in PC12 cells: potential role of Homer1. PLoS One 8:e55601. 10.1371/journal.pone.0055601 23383239 PMC3561331

[B35] Cheung K.-H. Shineman D. Müller M. Cárdenas C. Mei L. Yang J.. (2008). Mechanism of Ca^2+^ disruption in Alzheimer’s disease by presenilin regulation of InsP3 receptor channel gating. Neuron 58, 871–883. 10.1016/j.neuron.2008.04.015 18579078 PMC2495086

[B36] Czeredys M. Maciag F. Methner A. Kuznicki J. (2017). Tetrahydrocarbazoles decrease elevated SOCE in medium spiny neurons from transgenic YAC128 mice, a model of Huntington’s disease. Biochem. Biophys. Res. Commun. 483, 1194–1205. 10.1016/j.bbrc.2016.08.106 27553284

[B37] DeHaven W. I. Smyth J. T. Boyles R. R. Putney J. W. (2007). Calcium inhibition and calcium potentiation of Orai1, Orai2 and Orai3 calcium release-activated calcium channels. J. Biol. Chem. 282, 17548–17556. 10.1074/jbc.m611374200 17452328

[B38] DeJesus-Hernandez M. Mackenzie I. R. Boeve B. F. Boxer A. L. Baker M. Rutherford N. J.. (2011). Expanded GGGGCC hexanucleotide repeat in noncoding region of C9ORF72 causes chromosome 9p-linked FTD and ALS. Neuron 72, 245–256. 10.1016/j.neuron.2011.09.011 21944778 PMC3202986

[B39] Del Prete D. Checler F. Chami M. (2014). Ryanodine receptors: physiological function and deregulation in Alzheimer disease. Mol. Neurodegener. 9:21. 10.1186/1750-1326-9-21 24902695 PMC4063224

[B40] Demaurex N. Nunes P. (2016). The role of STIM and ORAI proteins in phagocytic immune cells. Am. J. Physiol. Cell Physiol. 310, C496–C508. 10.1152/ajpcell.00360.2015 26764049 PMC4824159

[B41] Di Giorgio F. P. Carrasco M. A. Siao M. C. Maniatis T. Eggan K. (2007). Non-cell autonomous effect of glia on motor neurons in an embryonic stem cell-based ALS model. Nat. Neurosci. 10, 608–614. 10.1038/nn1885 17435754 PMC3139463

[B42] Dirnagl U. Endres M. (2014). Found in translation: preclinical stroke research predicts human pathophysiology, clinical phenotypes and therapeutic outcomes. Stroke 45, 1510–1518. 10.1161/STROKEAHA.113.004075 24652307

[B43] Doody R. S. Raman R. Farlow M. Iwatsubo T. Vellas B. Joffe S.. (2013). A phase 3 trial of semagacestat for treatment of Alzheimer’s disease. N. Engl. J. Med. 369, 341–350. 10.1056/NEJMoa1210951 23883379

[B44] Duggan S. P. McCarthy J. V. (2016). Beyond γ-secretase activity: the multifunctional nature of presenilins in cell signalling pathways. Cell. Signal. 28, 1–11. 10.1016/j.cellsig.2015.10.006 26498858

[B45] Ferri K. F. Kroemer G. (2001). Organelle-specific initiation of cell death pathways. Nat. Cell Biol. 3, E255–E263. 10.1038/ncb1101-e255 11715037

[B46] Feske S. (2007). Calcium signalling in lymphocyte activation and disease. Nat. Rev. Immunol. 7, 690–702. 10.1038/nri2152 17703229

[B47] Feske S. (2011). Immunodeficiency due to defects in store-operated calcium entry. Ann. N Y Acad. Sci. 1238, 74–90. 10.1111/j.1749-6632.2011.06240.x 22129055 PMC3774594

[B48] Feske S. Giltnane J. Dolmetsch R. Staudt L. M. Rao A. (2001). Gene regulation mediated by calcium signals in T lymphocytes. Nat. Immunol. 2, 316–324. 10.1038/86318 11276202

[B49] Feske S. Gwack Y. Prakriya M. Srikanth S. Puppel S.-H. Tanasa B.. (2006). A mutation in Orai1 causes immune deficiency by abrogating CRAC channel function. Nature 441, 179–185. 10.1038/nature04702 16582901

[B50] Feske S. Prakriya M. Rao A. Lewis R. S. (2005). A severe defect in CRAC Ca^2+^ channel activation and altered K^+^ channel gating in T cells from immunodeficient patients. J. Exp. Med. 202, 651–662. 10.1084/jem.20050687 16147976 PMC2212870

[B51] Fjell A. M. McEvoy L. Holland D. Dale A. M. Walhovd K. B. Alzheimer’s Disease Neuroimaging Initiative. (2014). What is normal in normal aging? Effects of aging, amyloid and Alzheimer’s disease on the cerebral cortex and the hippocampus. Prog. Neurobiol. 117, 20–40. 10.1016/j.pneurobio.2014.02.004 24548606 PMC4343307

[B52] Fleming S. M. (2017). Mechanisms of gene-environment interactions in Parkinson’s disease. Curr. Environ. Health Rep. 4, 192–199. 10.1007/s40572-017-0143-2 28417442

[B53] Garcia-Alvarez G. Shetty M. S. Lu B. Yap K. A. F. Oh-Hora M. Sajikumar S.. (2015). Impaired spatial memory and enhanced long-term potentiation in mice with forebrain-specific ablation of the Stim genes. Front. Behav. Neurosci. 9:180. 10.3389/fnbeh.2015.00180 26236206 PMC4500926

[B54] Gibson G. E. Zhang H. Toral-Barza L. Szolosi S. Tofel-Grehl B. (1996). Calcium stores in cultured fibroblasts and their changes with Alzheimer’s disease. Biochim. Biophys. Acta 1316, 71–77. 10.1016/0925-4439(96)00002-6 8672553

[B55] Godyn J. Jonczyk J. Panek D. Malawska B. (2016). Therapeutic strategies for Alzheimer’s disease in clinical trials. Pharmacol. Rep. 68, 127–138. 10.1016/j.pharep.2015.07.006 26721364

[B56] Goel M. Sinkins W. G. Schilling W. P. (2002). Selective association of TRPC channel subunits in rat brain synaptosomes. J. Biol. Chem. 277, 48303–48310. 10.1074/jbc.M207882200 12377790

[B57] Golovina V. A. Platoshyn O. Bailey C. L. Wang J. Limsuwan A. Sweeney M.. (2001). Upregulated TRP and enhanced capacitative Ca^2+^ entry in human pulmonary artery myocytes during proliferation. Am. J. Physiol. Heart Circ. Physiol. 280, H746–H755. 10.1152/ajpheart.2001.280.2.h746 11158974

[B58] Green K. N. Demuro A. Akbari Y. Hitt B. D. Smith I. F. Parker I.. (2008). SERCA pump activity is physiologically regulated by presenilin and regulates amyloid β production. J. Cell Biol. 181, 1107–1116. 10.1083/jcb.200706171 18591429 PMC2442205

[B59] Haidet-Phillips A. M. Hester M. E. Miranda C. J. Meyer K. Braun L. Frakes A.. (2011). Astrocytes from familial and sporadic ALS patients are toxic to motor neurons. Nat. Biotechnol. 29, 824–828. 10.1038/nbt.1957 21832997 PMC3170425

[B60] Hawkins B. J. Irrinki K. M. Mallilankaraman K. Lien Y.-C. Wang Y. Bhanumathy C. D.. (2010). S-glutathionylation activates STIM1 and alters mitochondrial homeostasis. J. Cell Biol. 190, 391–405. 10.1083/jcb.201004152 20679432 PMC2922639

[B61] Haydon P. G. Carmignoto G. (2006). Astrocyte control of synaptic transmission and neurovascular coupling. Physiol. Rev. 86, 1009–1031. 10.1152/physrev.00049.2005 16816144

[B62] Hayrapetyan V. Rybalchenko V. Rybalchenko N. Koulen P. (2008). The N-terminus of presenilin-2 increases single channel activity of brain ryanodine receptors through direct protein-protein interaction. Cell Calcium 44, 507–518. 10.1016/j.ceca.2008.03.004 18440065

[B63] Hofmann T. Schaefer M. Schultz G. Gudermann T. (2002). Subunit composition of mammalian transient receptor potential channels in living cells. Proc. Natl. Acad. Sci. U S A 99, 7461–7466. 10.1073/pnas.102596199 12032305 PMC124253

[B64] Hort J. O’Brien J. T. Gainotti G. Pirttila T. Popescu B. O. Rektorova I.. (2010). EFNS guidelines for the diagnosis and management of Alzheimer’s disease. Eur. J. Neurol. 17, 1236–1248. 10.1111/j.1468-1331.2010.03040.x 20831773

[B65] Hoth M. Penner R. (1993). Calcium release-activated calcium current in rat mast cells. J. Physiol. 465, 359–386. 10.1113/jphysiol.1993.sp019681 8229840 PMC1175434

[B66] Huang G. N. Zeng W. Kim J. Y. Yuan J. P. Han L. Muallem S.. (2006). STIM1 carboxyl-terminus activates native SOC, I(crac) and TRPC1 channels. Nat. Cell Biol. 8, 1003–1010. 10.1038/ncb1454 16906149

[B67] Hulot J.-S. Fauconnier J. Ramanujam D. Chaanine A. Aubart F. Sassi Y.. (2011). Critical role for stromal interaction molecule 1 in cardiac hypertrophy. Circulation 124, 796–805. 10.1161/CIRCULATIONAHA.111.031229 21810664 PMC3428713

[B68] Ikeda M. Tsuno S. Sugiyama T. Hashimoto A. Yamoto K. Takeuchi K.. (2013). Ca^2+^ spiking activity caused by the activation of store-operated Ca^2+^ channels mediates TNF-α release from microglial cells under chronic purinergic stimulation. Biochim. Biophys. Acta 1833, 2573–2585. 10.1016/j.bbamcr.2013.06.022 23830920

[B69] Isegawa K. Hirooka Y. Katsuki M. Kishi T. Sunagawa K. (2014). Angiotensin II type 1 receptor expression in astrocytes is upregulated leading to increased mortality in mice with myocardial infarction-induced heart failure. Am. J. Physiol. Heart Circ. Physiol. 307, H1448–H1455. 10.1152/ajpheart.00462.2014 25217656

[B70] Ito E. Oka K. Etcheberrigaray R. Nelson T. J. McPhie D. L. Tofel-Grehl B.. (1994). Internal Ca^2+^ mobilization is altered in fibroblasts from patients with Alzheimer disease. Proc. Natl. Acad. Sci. U S A 91, 534–538. 10.1073/pnas.91.2.534 8290560 PMC42983

[B71] Jaworska A. Dzbek J. Styczynska M. Kuznicki J. (2013). Analysis of calcium homeostasis in fresh lymphocytes from patients with sporadic Alzheimer’s disease or mild cognitive impairment. Biochim. Biophys. Acta 1833, 1692–1699. 10.1016/j.bbamcr.2013.01.012 23354174

[B72] Jia S. Rodriguez M. Williams A. G. Yuan J. P. (2017). Homer binds to Orai1 and TRPC channels in the neointima and regulates vascular smooth muscle cell migration and proliferation. Sci. Rep. 7:5075. 10.1038/s41598-017-04747-w 28698564 PMC5506012

[B73] Karch C. M. Goate A. M. (2015). Alzheimer’s disease risk genes and mechanisms of disease pathogenesis. Biol. Psychiatry 77, 43–51. 10.1016/j.biopsych.2014.05.006 24951455 PMC4234692

[B74] Kaus A. Sareen D. (2015). ALS Patient stem cells for unveiling disease signatures of motoneuron susceptibility: perspectives on the deadly mitochondria, ER stress and calcium triad. Front. Cell. Neurosci. 9:448. 10.3389/fncel.2015.00448 26635528 PMC4652136

[B75] Kawamata H. Ng S. K. Diaz N. Burstein S. Morel L. Osgood A.. (2014). Abnormal intracellular calcium signaling and SNARE-dependent exocytosis contributes to SOD1G93A astrocyte-mediated toxicity in amyotrophic lateral sclerosis. J. Neurosci. 34, 2331–2348. 10.1523/jneurosci.2689-13.2014 24501372 PMC3913875

[B76] Khachaturian Z. S. (1989). Calcium, membranes, aging, and Alzheimer’s disease. Introduction and overview. Ann. N Y Acad. Sci. 568, 1–4. 10.1111/j.1749-6632.1989.tb12485.x 2629579

[B77] Kim S. D. Fung V. S. C. (2014). An update on Huntington’s disease. Curr. Opin. Neurol. 27, 477–483. 10.1097/WCO.0000000000000116 24978638

[B78] Korkotian E. Oni-Biton E. Segal M. (2017). The role of the store-operated calcium entry channel Orai1 in cultured rat hippocampal synapse formation and plasticity. J. Physiol. 595, 125–140. 10.1113/jp272645 27393042 PMC5199748

[B79] Kraft R. (2015). STIM and ORAI proteins in the nervous system. Channels 9, 245–252. 10.1080/19336950.2015.1071747 26218135 PMC4826113

[B80] Kuchibhotla K. V. Goldman S. T. Lattarulo C. R. Wu H.-Y. Hyman B. T. Bacskai B. J. (2008). Aβ plaques lead to aberrant regulation of calcium homeostasis *in vivo* resulting in structural and functional disruption of neuronal networks. Neuron 59, 214–225. 10.1016/j.neuron.2008.06.008 18667150 PMC2578820

[B81] Kumar V. S. S. Gopalakrishnan A. Naziroglu M. Rajanikant G. K. (2014). Calcium ion—the key player in cerebral ischemia. Curr. Med. Chem. 21, 2065–2275. 10.2174/0929867321666131228204246 24372212

[B82] Lacampagne A. Liu X. Reiken S. Bussiere R. Meli A. C. Lauritzen I.. (2017). Post-translational remodeling of ryanodine receptor induces calcium leak leading to Alzheimer’s disease-like pathologies and cognitive deficits. Acta Neuropathol. 134, 749–767. 10.1007/s00401-017-1733-7 28631094

[B83] Lacruz R. S. Feske S. (2015). Diseases caused by mutations in ORAI1 and STIM1. Ann. N Y Acad. Sci. 1356, 45–79. 10.1111/nyas.12938 26469693 PMC4692058

[B84] Lee M. Jantaratnotai N. McGeer E. McLarnon J. G. McGeer P. L. (2011). Mg^2+^ ions reduce microglial and THP-1 cell neurotoxicity by inhibiting Ca^2+^ entry through purinergic channels. Brain Res. 1369, 21–35. 10.1016/j.brainres.2010.10.084 21040713

[B85] Lein E. S. Hawrylycz M. J. Ao N. Ayres M. Bensinger A. Bernard A.. (2007). Genome-wide atlas of gene expression in the adult mouse brain. Nature 445, 168–176. 10.1038/nature05453 17151600

[B86] Lessard C. B. Lussier M. P. Cayouette S. Bourque G. Boulay G. (2005). The overexpression of presenilin2 and Alzheimer’s-disease-linked presenilin2 variants influences TRPC6-enhanced Ca^2+^ entry into HEK293 cells. Cell. Signal. 17, 437–445. 10.1016/j.cellsig.2004.09.005 15601622

[B87] Lewis R. S. Cahalan M. D. (1995). Potassium and calcium channels in lymphocytes. Annu. Rev. Immunol. 13, 623–653. 10.1146/annurev.iy.13.040195.003203 7612237

[B89] Li X. Chen W. Zhang L. Liu W. Fei Z. (2013). Inhibition of store-operated calcium entry attenuates MPP^+^-induced oxidative stress via preservation of mitochondrial function in PC12 cells: involvement of homer1a. PLoS One 8:e83638. 10.1371/journal.pone.0083638 24358303 PMC3866123

[B88] Li B. Xiao L. Wang Z. Y. Zheng P. S. (2014). Knockdown of STIM1 inhibits 6-hydroxydopamine-induced oxidative stress through attenuating calcium-dependent ER stress and mitochondrial dysfunction in undifferentiated PC12 cells. Free Radic. Res. 48, 758–768. 10.3109/10715762.2014.905687 24720513

[B90] Liao Y. Erxleben C. Yildirim E. Abramowitz J. Armstrong D. L. Birnbaumer L. (2007). Orai proteins interact with TRPC channels and confer responsiveness to store depletion. Proc. Natl. Acad. Sci. U S A 104, 4682–4687. 10.1073/pnas.0611692104 17360584 PMC1838661

[B91] Lind B. L. Jessen S. B. Lønstrup M. Joséphine C. Bonvento G. Lauritzen M. (2018). Fast Ca^2+^ responses in astrocyte end-feet and neurovascular coupling in mice. Glia 66, 348–358. 10.1002/glia.23246 29058353

[B92] Linde C. I. Baryshnikov S. G. Mazzocco-Spezzia A. Golovina V. A. (2011). Dysregulation of Ca^2+^ signaling in astrocytes from mice lacking amyloid precursor protein. Am. J. Physiol. Cell Physiol. 300, C1502–C1512. 10.1152/ajpcell.00379.2010 21368296 PMC3118622

[B93] Liou J. Kim M. L. Heo W. D. Jones J. T. Myers J. W. Ferrell J. E.. (2005). STIM is a Ca^2+^ sensor essential for Ca^2+^-store-depletion-triggered Ca^2+^ influx. Curr. Biol. 15, 1235–1241. 10.1016/j.cub.2005.05.055 16005298 PMC3186072

[B94] Lis A. Peinelt C. Beck A. Parvez S. Monteilh-Zoller M. Fleig A.. (2007). CRACM1, CRACM2 and CRACM3 are store-operated Ca^2+^ channels with distinct functional properties. Curr. Biol. 17, 794–800. 10.1016/j.cub.2007.03.065 17442569 PMC5663639

[B96] Liu X. Bandyopadhyay B. C. Singh B. B. Groschner K. Ambudkar I. S. (2005). Molecular analysis of a store-operated and 2-acetyl-sn-glycerol-sensitive non-selective cation channel. Heteromeric assembly of TRPC1-TRPC3. J. Biol. Chem. 280, 21600–21606. 10.1074/jbc.c400492200 15834157

[B97] Liu X. Singh B. B. Ambudkar I. S. (2003). TRPC1 is required for functional store-operated Ca ^2+^ channels. Role of acidic amino acid residues in the S5-S6 region. J. Biol. Chem. 278, 11337–11343. 10.1074/jbc.M213271200 12536150

[B95] Liu D. Y. Thilo F. Scholze A. Wittstock A. Zhao Z. G. Harteneck C.. (2007). Increased store-operated and 1-oleoyl-2-acetyl-sn-glycerol-induced calcium influx in monocytes is mediated by transient receptor potential canonical channels in human essential hypertension. J. Hypertens. 25, 799–808. 10.1097/hjh.0b013e32803cae2b 17351372

[B98] Llano I. González J. Caputo C. Lai F. A. Blayney L. M. Tan Y. P.. (2000). Presynaptic calcium stores underlie large-amplitude miniature IPSCs and spontaneous calcium transients. Nat. Neurosci. 3, 1256–1265. 10.1038/81781 11100146

[B99] Lopez J. J. Albarran L. Gómez L. J. Smani T. Salido G. M. Rosado J. A. (2016). Molecular modulators of store-operated calcium entry. Biochim. Biophys. Acta 1863, 2037–2043. 10.1016/j.bbamcr.2016.04.024 27130253

[B100] Lu L. Hogan-Cann A. D. Globa A. K. Lu P. Nagy J. I. Bamji S. X.. (2017). Astrocytes drive cortical vasodilatory signaling by activating endothelial NMDA receptors. J. Cereb. Blood Flow Metab. [Epub ahead of print]. 10.1177/0271678x17734100 29072857 PMC6421257

[B101] Luik R. M. Wu M. M. Buchanan J. Lewis R. S. (2006). The elementary unit of store-operated Ca^2+^ entry: local activation of CRAC channels by STIM1 at ER-plasma membrane junctions. J. Cell Biol. 174, 815–825. 10.1083/jcb.200604015 16966423 PMC2064336

[B102] Ma G. Wei M. He L. Liu C. Wu B. Zhang S. L.. (2015). Inside-out Ca^2+^ signalling prompted by STIM1 conformational switch. Nat. Commun. 6:7826. 10.1038/ncomms8826 26184105 PMC4509486

[B103] Majewski L. Kuznicki J. (2015). SOCE in neurons: signaling or just refilling? Biochim. Biophys. Acta 1853, 1940–1952. 10.1016/j.bbamcr.2015.01.019 25646572

[B104] McColgan P. Tabrizi S. J. (2018). Huntington’s disease: a clinical review. Eur. J. Neurol. 25, 24–34. 10.1111/ene.13413 28817209

[B105] McKhann G. M. Knopman D. S. Chertkow H. Hyman B. T. Jack C. R. Kawas C. H.. (2011). The diagnosis of dementia due to Alzheimer’s disease: recommendations from the National Institute on Aging-Alzheimer’s Association workgroups on diagnostic guidelines for Alzheimer’s disease. Alzheimers Dement. 7, 263–269. 10.1016/j.jalz.2011.03.005 21514250 PMC3312024

[B106] Michaelis M. Nieswandt B. Stegner D. Eilers J. Kraft R. (2015). STIM1, STIM2 and Orai1 regulate store-operated calcium entry and purinergic activation of microglia. Glia 63, 652–663. 10.1002/glia.22775 25471906

[B107] Mieyal J. J. Gallogly M. M. Qanungo S. Sabens E. A. Shelton M. D. (2008). Molecular mechanisms and clinical implications of reversible protein S -glutathionylation. Antioxid. Redox Signal. 10, 1941–1988. 10.1089/ars.2008.2089 18774901 PMC2774718

[B108] Mishra A. Reynolds J. P. Chen Y. Gourine A. V. Rusakov D. A. Attwell D. (2016). Astrocytes mediate neurovascular signaling to capillary pericytes but not to arterioles. Nat. Neurosci. 19, 1619–1627. 10.1038/nn.4428 27775719 PMC5131849

[B109] Moccia F. Tanzi F. Munaron L. (2014). Endothelial remodelling and intracellular calcium machinery. Curr. Mol. Med. 14, 457–480. 10.2174/1566524013666131118113410 24236452

[B110] Moccia F. Zuccolo E. Soda T. Tanzi F. Guerra G. Mapelli L.. (2015). Stim and Orai proteins in neuronal Ca^2+^ signaling and excitability. Front. Cell. Neurosci. 9:153. 10.3389/fncel.2015.00153 25964739 PMC4408853

[B111] Müller W. Connor J. A. (1991). Dendritic spines as individual neuronal compartments for synaptic Ca^2+^ responses. Nature 354, 73–76. 10.1038/354073a0 1682815

[B112] Nagai M. Re D. B. Nagata T. Chalazonitis A. Jessell T. M. Wichterle H.. (2007). Astrocytes expressing ALS-linked mutated SOD1 release factors selectively toxic to motor neurons. Nat. Neurosci. 10, 615–622. 10.1038/nn1876 17435755 PMC3799799

[B113] Nelson O. Supnet C. Tolia A. Horré K. De Strooper B. Bezprozvanny I. (2011). Mutagenesis mapping of the presenilin 1 calcium leak conductance pore. J. Biol. Chem. 286, 22339–22347. 10.1074/jbc.m111.243063 21531718 PMC3121381

[B114] Nelson O. Tu H. Lei T. Bentahir M. de Strooper B. Bezprozvanny I. (2007). Familial Alzheimer disease-linked mutations specifically disrupt Ca^2+^ leak function of presenilin 1. J. Clin. Invest. 117, 1230–1239. 10.1172/jci30447 17431506 PMC1847535

[B115] Neuner S. M. Wilmott L. A. Hoffmann B. R. Mozhui K. Kaczorowski C. C. (2017). Hippocampal proteomics defines pathways associated with memory decline and resilience in normal aging and Alzheimer’s disease mouse models. Behav. Brain Res. 322, 288–298. 10.1016/j.bbr.2016.06.002 27265785 PMC5135662

[B116] Neuner S. M. Wilmott L. A. Hope K. A. Hoffmann B. Chong J. A. Abramowitz J.. (2015). TRPC3 channels critically regulate hippocampal excitability and contextual fear memory. Behav. Brain Res. 281, 69–77. 10.1016/j.bbr.2014.12.018 25513972 PMC4677051

[B117] Ohara H. Nabika T. (2016). A nonsense mutation of Stim1 identified in stroke-prone spontaneously hypertensive rats decreased the store-operated calcium entry in astrocytes. Biochem. Biophys. Res. Commun. 476, 406–411. 10.1016/j.bbrc.2016.05.134 27237974

[B118] Oh-Hora M. Yamashita M. Hogan P. G. Sharma S. Lamperti E. Chung W.. (2008). Dual functions for the endoplasmic reticulum calcium sensors STIM1 and STIM2 in T cell activation and tolerance. Nat. Immunol. 9, 432–443. 10.1038/ni1574 18327260 PMC2737533

[B119] Ong H. L. de Souza L. B. Ambudkar I. S. (2016). Role of TRPC channels in store-operated calcium entry. Adv. Exp. Med. Biol. 898, 87–109. 10.1007/978-3-319-26974-0_5 27161226

[B120] Oulès B. Del Prete D. Greco B. Zhang X. Lauritzen I. Sevalle J.. (2012). Ryanodine receptor blockade reduces amyloid-β load and memory impairments in Tg2576 mouse model of Alzheimer disease. J. Neurosci. 32, 11820–11834. 10.1523/jneurosci.0875-12.2012 22915123 PMC3458216

[B121] Pannaccione A. Secondo A. Molinaro P. D’Avanzo C. Cantile M. Esposito A.. (2012). A new concept: A β1–42 generates a hyperfunctional proteolytic NCX3 fragment that delays caspase-12 activation and neuronal death. J. Neurosci. 32, 10609–10617. 10.1523/JNEUROSCI.6429-11.2012 22855810 PMC6621392

[B122] Parekh A. B. (2010). Store-operated CRAC channels: function in health and disease. Nat. Rev. Drug Discov. 9, 399–410. 10.1038/nrd3136 20395953

[B123] Parekh A. B. Penner R. (1997). Store depletion and calcium influx. Physiol. Rev. 77, 901–930. 10.1152/physrev.1997.77.4.901 9354808

[B124] Pareyson D. Piscosquito G. Moroni I. Salsano E. Zeviani M. (2013). Peripheral neuropathy in mitochondrial disorders. Lancet Neurol. 12, 1011–1024. 10.1016/S1474-4422(13)70158-3 24050734

[B125] Pathak T. Agrawal T. Richhariya S. Sadaf S. Hasan G. (2015). Store-operated calcium entry through orai is required for transcriptional maturation of the flight circuit in *Drosophila*. J. Neurosci. 35, 13784–13799. 10.1523/JNEUROSCI.1680-15.2015 26446229 PMC6605383

[B126] Pchitskaya E. Popugaeva E. Bezprozvanny I. (2018). Calcium signaling and molecular mechanisms underlying neurodegenerative diseases. Cell Calcium 70, 87–94. 10.1016/j.ceca.2017.06.008 28728834 PMC5748019

[B127] Pedata F. Dettori I. Coppi E. Melani A. Fusco I. Corradetti R.. (2016). Purinergic signalling in brain ischemia. Neuropharmacology 104, 105–130. 10.1016/j.neuropharm.2015.11.007 26581499

[B128] Peinelt C. Vig M. Koomoa D. L. Beck A. Nadler M. J. S. Koblan-Huberson M.. (2006). Amplification of CRAC current by STIM1 and CRACM1 (Orai1). Nat. Cell Biol. 8, 771–773. 10.1038/ncb1435 16733527 PMC5685802

[B129] Peng J. Liang G. Inan S. Wu Z. Joseph D. J. Meng Q.. (2012). Dantrolene ameliorates cognitive decline and neuropathology in Alzheimer triple transgenic mice. Neurosci. Lett. 516, 274–279. 10.1016/j.neulet.2012.04.008 22516463 PMC3351794

[B130] Pinton P. Rizzuto R. (2006). Bcl-2 and Ca^2+^ homeostasis in the endoplasmic reticulum. Cell Death Differ. 13, 1409–1418. 10.1038/sj.cdd.4401960 16729032

[B131] Pla-Martín D. Rueda C. B. Estela A. Sánchez-Piris M. González-Sánchez P. Traba J.. (2013). Silencing of the Charcot-Marie-Tooth disease-associated gene GDAP1 induces abnormal mitochondrial distribution and affects Ca^2+^ homeostasis by reducing store-operated Ca^2+^ entry. Neurobiol. Dis. 55, 140–151. 10.1016/j.nbd.2013.03.010 23542510

[B132] Poon H. F. Hensley K. Thongboonkerd V. Merchant M. L. Lynn B. C. Pierce W. M.. (2005). Redox proteomics analysis of oxidatively modified proteins in G93A-SOD1 transgenic mice—a model of familial amyotrophic lateral sclerosis. Free Radic. Biol. Med. 39, 453–462. 10.1016/j.freeradbiomed.2005.03.030 16043017

[B133] Popugaeva E. Pchitskaya E. Speshilova A. Alexandrov S. Zhang H. Vlasova O.. (2015). STIM2 protects hippocampal mushroom spines from amyloid synaptotoxicity. Mol. Neurodegener. 10:37. 10.1186/s13024-015-0034-7 26275606 PMC4536802

[B134] Potier M. Gonzalez J. C. Motiani R. K. Abdullaev I. F. Bisaillon J. M. Singer H. A.. (2009). Evidence for STIM1- and Orai1-dependent store-operated calcium influx through ICRAC in vascular smooth muscle cells: role in proliferation and migration. FASEB J. 23, 2425–2437. 10.1096/fj.09-131128 19364762 PMC2717784

[B135] Prakriya M. Feske S. Gwack Y. Srikanth S. Rao A. Hogan P. G. (2006). Orai1 is an essential pore subunit of the CRAC channel. Nature 443, 230–233. 10.1038/nature05122 16921383

[B136] Putney J. W. (2017). Forms and functions of store-operated calcium entry mediators, STIM and Orai. Adv. Biol. Regul. [Epub ahead of print]. 10.1016/j.jbior.2017.11.006 29217255 PMC5955777

[B137] Raymond L. A. (2017). Striatal synaptic dysfunction and altered calcium regulation in Huntington disease. Biochem. Biophys. Res. Commun. 483, 1051–1062. 10.1016/j.bbrc.2016.07.058 27423394

[B138] Renton A. E. Majounie E. Waite A. Simón-Sánchez J. Rollinson S. Gibbs J. R.. (2011). A hexanucleotide repeat expansion in C9ORF72 is the cause of chromosome 9p21-linked ALS-FTD. Neuron 72, 257–268. 10.1016/j.neuron.2011.09.010 21944779 PMC3200438

[B139] Ronco V. Grolla A. A. Glasnov T. N. Canonico P. L. Verkhratsky A. Genazzani A. A.. (2014). Differential deregulation of astrocytic calcium signalling by amyloid-β, TNFα, IL-1β and LPS. Cell Calcium 55, 219–229. 10.1016/j.ceca.2014.02.016 24656753

[B140] Roos J. DiGregorio P. J. Yeromin A. V. Ohlsen K. Lioudyno M. Zhang S.. (2005). STIM1, an essential and conserved component of store-operated Ca^2+^ channel function. J. Cell Biol. 169, 435–445. 10.1083/jcb.200502019 15866891 PMC2171946

[B141] Rosado J. A. (2016). Calcium entry pathways in non-excitable cells. Adv. Exp. Med. Biol. 898, vii–viii. 10.1007/978-3-319-26974-0 27540620

[B142] Rosen D. R. Siddique T. Patterson D. Figlewicz D. A. Sapp P. Hentati A.. (1993). Mutations in Cu/Zn superoxide dismutase gene are associated with familial amyotrophic lateral sclerosis. Nature 362, 59–62. 10.1136/jmg.30.6.532-b 8446170

[B143] Ryskamp D. Wu J. Geva M. Kusko R. Grossman I. Hayden M.. (2017). The sigma-1 receptor mediates the beneficial effects of pridopidine in a mouse model of Huntington disease. Neurobiol. Dis. 97, 46–59. 10.1016/j.nbd.2016.10.006 27818324 PMC5214572

[B144] Saliba Y. Keck M. Marchand A. Atassi F. Ouillé A. Cazorla O.. (2015). Emergence of Orai3 activity during cardiac hypertrophy. Cardiovasc. Res. 105, 248–259. 10.1093/cvr/cvu207 25213556 PMC4351368

[B145] Scheltens P. Blennow K. Breteler M. M. B. de Strooper B. Frisoni G. B. Salloway S.. (2016). Alzheimer’s disease. Lancet 388, 505–517. 10.1016/S0140-6736(15)01124-1 26921134

[B146] Selkoe D. J. Hardy J. (2016). The amyloid hypothesis of Alzheimer’s disease at 25 years. EMBO Mol. Med. 8, 595–608. 10.15252/emmm.201606210 27025652 PMC4888851

[B147] Selvaraj S. Sun Y. Watt J. A. Wang S. Lei S. Birnbaumer L.. (2012). Neurotoxin-induced ER stress in mouse dopaminergic neurons involves downregulation of TRPC1 and inhibition of AKT/mTOR signaling. J. Clin. Invest. 122, 1354–1367. 10.1172/JCI61332 22446186 PMC3314472

[B148] Serrano-Pozo A. Frosch M. P. Masliah E. Hyman B. T. (2011). Neuropathological alterations in Alzheimer disease. Cold Spring Harb. Perspect. Med. 1:a006189. 10.1101/cshperspect.a006189 22229116 PMC3234452

[B149] Shilling D. Mak D. O. Kang D. E. Foskett J. K. (2012). Lack of evidence for presenilins as endoplasmic reticulum Ca^2+^ leak channels. J. Biol. Chem. 287, 10933–10944. 10.1074/jbc.M111.300491 22311977 PMC3322867

[B150] Shilling D. Müller M. Takano H. Mak D.-O. Abel T. Coulter D. A.. (2014). Suppression of InsP3 receptor-Mediated Ca^2+^ signaling alleviates mutant presenilin-linked familial Alzheimer’s disease pathogenesis. J. Neurosci. 34, 6910–6923. 10.1523/jneurosci.5441-13.2014 24828645 PMC4019804

[B151] Sirabella R. Secondo A. Pannaccione A. Scorziello A. Valsecchi V. Adornetto A.. (2009). Anoxia-induced NF-κB-dependent upregulation of NCX1 contributes to Ca^2+^ refilling into endoplasmic reticulum in cortical neurons. Stroke 40, 922–929. 10.1161/strokeaha.108.531962 19164785

[B152] Smith I. F. Boyle J. P. Kang P. Rome S. Pearson H. A. Peers C. (2005). Hypoxic regulation of Ca^2+^ signaling in cultured rat astrocytes. Glia 49, 153–157. 10.1002/glia.20083 15390111

[B153] Soboloff J. Spassova M. A. Hewavitharana T. He L.-P. Xu W. Johnstone L. S.. (2006). STIM2 is an inhibitor of STIM1-Mediated store-operated Ca^2+^ entry. Curr. Biol. 16, 1465–1470. 10.1016/j.cub.2006.05.051 16860747

[B154] Sodero A. O. Vriens J. Ghosh D. Stegner D. Brachet A. Pallotto M.. (2012). Cholesterol loss during glutamate-mediated excitotoxicity. EMBO J. 31, 1764–1773. 10.1038/emboj.2012.31 22343944 PMC3321209

[B155] Stathopulos P. B. Zheng L. Ikura M. (2009). Stromal interaction molecule (STIM) 1 and STIM2 calcium sensing regions exhibit distinct unfolding and oligomerization kinetics. J. Biol. Chem. 284, 728–732. 10.1074/jbc.c800178200 19019825

[B156] Stefani I. C. Wright D. Polizzi K. M. Kontoravdi C. (2012). The role of ER stress-induced apoptosis in neurodegeneration. Curr. Alzheimer Res. 9, 373–387. 10.2174/156720512800107618 22299619

[B157] Strübing C. Krapivinsky G. Krapivinsky L. Clapham D. E. (2003). Formation of novel TRPC channels by complex subunit interactions in embryonic brain. J. Biol. Chem. 278, 39014–39019. 10.1074/jbc.m306705200 12857742

[B158] Stutzmann G. E. Smith I. Caccamo A. Oddo S. Laferla F. M. Parker I. (2006). Enhanced ryanodine receptor recruitment contributes to Ca^2+^ disruptions in young, adult and aged Alzheimer’s disease mice. J. Neurosci. 26, 5180–5189. 10.1523/jneurosci.0739-06.2006 16687509 PMC6674246

[B159] Stutzmann G. E. Smith I. Caccamo A. Oddo S. Parker I. Laferla F. (2007). Enhanced ryanodine-mediated calcium release in mutant PS1-expressing Alzheimer’s mouse models. Ann. N Y Acad. Sci. 1097, 265–277. 10.1196/annals.1379.025 17413028

[B160] Sulger J. Dumais-Huber C. Zerfass R. Henn F. A. Aldenhoff J. B. (1999). The calcium response of human T lymphocytes is decreased in aging but increased in Alzheimer’s dementia. Biol. Psychiatry 45, 737–742. 10.1016/s0006-3223(98)00218-2 10188003

[B161] Sun S. Zhang H. Liu J. Popugaeva E. Xu N.-J. Feske S.. (2014). Reduced synaptic STIM2 expression and impaired store-operated calcium entry cause destabilization of mature spines in mutant presenilin mice. Neuron 82, 79–93. 10.1016/j.neuron.2014.02.019 24698269 PMC4007018

[B162] Sun Y. Zhang H. Selvaraj S. Sukumaran P. Lei S. Birnbaumer L.. (2017). Inhibition of L-Type Ca^2+^ channels by TRPC1-STIM1 complex is essential for the protection of dopaminergic neurons. J. Neurosci. 37, 3364–3377. 10.1523/jneurosci.3010-16.2017 28258168 PMC5373123

[B163] Sundivakkam P. C. Freichel M. Singh V. Yuan J. P. Vogel S. M. Flockerzi V.. (2012). The Ca^2+^ sensor stromal interaction molecule 1 (STIM1) is necessary and sufficient for the store-operated Ca^2+^ entry function of transient receptor potential canonical (TRPC) 1 and 4 channels in endothelial cells. Mol. Pharmacol. 81, 510–526. 10.1124/mol.111.074658 22210847 PMC3310414

[B164] Tang T.-S. Guo C. Wang H. Chen X. Bezprozvanny I. (2009). Neuroprotective effects of inositol 1,4,5-trisphosphate receptor C-terminal fragment in a Huntington’s disease mouse model. J. Neurosci. 29, 1257–1266. 10.1523/JNEUROSCI.4411-08.2009 19193873 PMC2768402

[B165] Tang T.-S. Slow E. Lupu V. Stavrovskaya I. G. Sugimori M. Llinás R.. (2005). Disturbed Ca^2+^ signaling and apoptosis of medium spiny neurons in Huntington’s disease. Proc. Natl. Acad. Sci. U S A 102, 2602–2607. 10.1073/pnas.0409402102 15695335 PMC548984

[B166] Tong B. C.-K. Lee C. S.-K. Cheng W.-H. Lai K.-O. Foskett J. K. Cheung K.-H. (2016). Familial Alzheimer’s disease-associated presenilin 1 mutants promote γ-secretase cleavage of STIM1 to impair store-operated Ca^2+^ entry. Sci. Signal. 9:ra89. 10.1126/scisignal.aaf1371 27601731 PMC5384262

[B167] Trepakova E. S. Gericke M. Hirakawa Y. Weisbrod R. M. Cohen R. A. Bolotina V. M. (2001). Properties of a native cation channel activated by Ca^2+^ store depletion in vascular smooth muscle cells. J. Biol. Chem. 276, 7782–7790. 10.1074/jbc.m010104200 11113149

[B168] Uhlén M. Fagerberg L. Hallstrom B. M. Lindskog C. Oksvold P. Mardinoglu A.. (2015). Proteomics. Tissue-based map of the human proteome. Science 347:1260419. 10.1126/science.1260419 25613900

[B169] Vaeth M. Yang J. Yamashita M. Zee I. Eckstein M. Knosp C.. (2017). ORAI2 modulates store-operated calcium entry and T cell-mediated immunity. Nat. Commun. 8:14714. 10.1038/ncomms14714 28294127 PMC5355949

[B170] van Kruchten R. Braun A. Feijge M. A. H. Kuijpers M. J. E. Rivera-Galdos R. Kraft P.. (2012). Antithrombotic potential of blockers of store-operated calcium channels in platelets. Arterioscler. Thromb. Vasc. Biol. 32, 1717–1723. 10.1161/atvbaha.111.243907 22580895

[B171] Varga-Szabo D. Braun A. Kleinschnitz C. Bender M. Pleines I. Pham M.. (2008). The calcium sensor STIM1 is an essential mediator of arterial thrombosis and ischemic brain infarction. J. Exp. Med. 205, 1583–1591. 10.1084/jem.20080302 18559454 PMC2442636

[B172] Venkatachalam K. Montell C. (2007). TRP channels. Annu. Rev. Biochem. 76, 387–417. 10.1146/annurev.biochem.75.103004.142819 17579562 PMC4196875

[B173] Vig M. Peinelt C. Beck A. Koomoa D. L. Rabah D. Koblan-Huberson M.. (2006). CRACM1 is a plasma membrane protein essential for store-operated Ca^2+^ entry. Science 312, 1220–1223. 10.1126/science.1127883 16645049 PMC5685805

[B175] Vigont V. Kolobkova Y. Skopin A. Zimina O. Zenin V. Glushankova L.. (2015). Both Orai1 and TRPC1 are involved in excessive store-operated calcium entry in striatal neurons expressing mutant Huntingtin exon 1. Front. Physiol. 6:337. 10.3389/fphys.2015.00337 26635623 PMC4656824

[B174] Vigont V. A. Zimina O. A. Glushankova L. N. Kolobkova J. A. Ryazantseva M. A. Mozhayeva G. N.. (2014). STIM1 Protein activates store-operated calcium channels in cellular model of Huntington’s disease. Acta Naturae 6, 40–47. 25558393 PMC4273090

[B176] Viscomi M. T. Florenzano F. Latini L. Amantea D. Bernardi G. Molinari M. (2008). Methylprednisolone treatment delays remote cell death after focal brain lesion. Neuroscience 154, 1267–1282. 10.1016/j.neuroscience.2008.04.024 18550289

[B177] Wang J. Lu R. Yang J. Li H. He Z. Jing N.. (2015). TRPC6 specifically interacts with APP to inhibit its cleavage by γ-secretase and reduce Aβ production. Nat. Commun. 6:8876. 10.1038/ncomms9876 26581893 PMC4696454

[B178] Wang X. Zhou X. Li G. Zhang Y. Wu Y. Song W. (2017). Modifications and trafficking of APP in the pathogenesis of Alzheimer’s disease. Front. Mol. Neurosci. 10:294. 10.3389/fnmol.2017.00294 28966576 PMC5605621

[B179] White C. (2017). The Regulation of tumor cell invasion and metastasis by endoplasmic reticulum-to-mitochondrial Ca^2+^ transfer. Front. Oncol. 7:171. 10.3389/fonc.2017.00171 28848710 PMC5554129

[B180] Williams R. T. Manji S. S. Parker N. J. Hancock M. S. Van Stekelenburg L. Eid J. P.. (2001). Identification and characterization of the STIM (stromal interaction molecule) gene family: coding for a novel class of transmembrane proteins. Biochem. J. 357, 673–685. 10.1042/0264-6021:3570673 11463338 PMC1221997

[B181] Wu J. Ryskamp D. A. Liang X. Egorova P. Zakharova O. Hung G.. (2016). Enhanced store-operated calcium entry leads to striatal synaptic loss in a Huntington’s disease mouse model. J. Neurosci. 36, 125–141. 10.1523/jneurosci.1038-15.2016 26740655 PMC4701955

[B182] Wu J. Shih H.-P. Vigont V. Hrdlicka L. Diggins L. Singh C.. (2011). Neuronal store-operated calcium entry pathway as a novel therapeutic target for Huntington’s disease treatment. Chem. Biol. 18, 777–793. 10.1016/j.chembiol.2011.04.012 21700213 PMC3124661

[B183] Wu X. Zagranichnaya T. K. Gurda G. T. Eves E. M. Villereal M. L. (2004). A TRPC1/TRPC3-mediated increase in store-operated calcium entry is required for differentiation of H19–7 hippocampal neuronal cells. J. Biol. Chem. 279, 43392–43402. 10.1074/jbc.m408959200 15297455

[B184] Yang S. Zhang J. J. Huang X.-Y. (2009). Orai1 and STIM1 are critical for breast tumor cell migration and metastasis. Cancer Cell 15, 124–134. 10.1016/j.ccr.2008.12.019 19185847

[B200] Zagranichnaya T. K. Wu X. Villereal M. L. (2005). Endogenous TRPC1, TRPC3, and TRPC7 proteins combine to form native store-operated channels in HEK-293 cells. J. Biol. Chem. 280, 29559–29569. 10.1074/jbc.M505842200 15972814

[B185] Zeiger W. Vetrivel K. S. Buggia-Prévot V. Nguyen P. D. Wagner S. L. Villereal M. L.. (2013). Ca^2+^ influx through store-operated Ca^2+^ channels reduces Alzheimer disease β-amyloid peptide secretion. J. Biol. Chem. 288, 26955–26966. 10.1074/jbc.m113.473355 23902769 PMC3772244

[B192] Zhang W. Halligan K. E. Zhang X. Bisaillon J. M. Gonzalez-Cobos J. C. Motiani R. K.. (2011). Orai1-mediated I (CRAC) is essential for neointima formation after vascular injury. Circ. Res. 109, 534–542. 10.1161/circresaha.111.246777 21737791 PMC3164514

[B186] Zhang H. Liu J. Sun S. Pchitskaya E. Popugaeva E. Bezprozvanny I. (2015a). Calcium signaling, excitability and synaptic plasticity defects in a mouse model of Alzheimer’s disease. J. Alzheimers Dis. 45, 561–580. 10.3233/JAD-142427 25589721 PMC4814213

[B189] Zhang H. Wu L. Pchitskaya E. Zakharova O. Saito T. Saido T.. (2015b). Neuronal store-operated calcium entry and mushroom spine loss in amyloid precursor protein knock-in mouse model of Alzheimer’s disease. J. Neurosci. 35, 13275–13286. 10.1523/jneurosci.1034-15.2015 26424877 PMC4588605

[B187] Zhang H. Sun S. Herreman A. De Strooper B. Bezprozvanny I. (2010). Role of presenilins in neuronal calcium homeostasis. J. Neurosci. 30, 8566–8580. 10.1523/JNEUROSCI.1554-10.2010 20573903 PMC2906098

[B188] Zhang H. Sun S. Wu L. Pchitskaya E. Zakharova O. Fon Tacer K.. (2016). Store-operated calcium channel complex in postsynaptic spines: a new therapeutic target for Alzheimer’s disease treatment. J. Neurosci. 36, 11837–11850. 10.1523/jneurosci.1188-16.2016 27881772 PMC5125243

[B190] Zhang M. Song J.-N. Wu Y. Zhao Y.-L. Pang H.-G. Fu Z.-F.. (2014). Suppression of STIM1 in the early stage after global ischemia attenuates the injury of delayed neuronal death by inhibiting store-operated calcium entry-induced apoptosis in rats. Neuroreport 25, 507–513. 10.1097/wnr.0000000000000127 24509424

[B191] Zhang S. L. Yu Y. Roos J. Kozak J. A. Deerinck T. J. Ellisman M. H.. (2005). STIM1 is a Ca^2+^ sensor that activates CRAC channels and migrates from the Ca^2+^ store to the plasma membrane. Nature 437, 902–905. 10.1038/nature04147 16208375 PMC1618826

[B193] Zhou J. Du W. Zhou K. Tai Y. Yao H. Jia Y.. (2008). Critical role of TRPC6 channels in the formation of excitatory synapses. Nat. Neurosci. 11, 741–743. 10.1038/nn.2127 18516035

[B194] Zhou Q. Yen A. Rymarczyk G. Asai H. Trengrove C. Aziz N.. (2016). Impairment of PARK14-dependent Ca^2+^ signalling is a novel determinant of Parkinson’s disease. Nat. Commun. 7:10332. 10.1038/ncomms10332 26755131 PMC4729940

